# Discriminative Capabilities of Eye Gaze Measures for Cognitive Load Evaluation in a Driving Simulation Task

**DOI:** 10.3390/jemr19010001

**Published:** 2025-12-24

**Authors:** Anastasiia Bakhchina, Karina Arutyunova, Evgenii Burashnikov, Anastasiya Filatova, Andrei Filimonov, Ivan Shishalov

**Affiliations:** Cognitive Systems Lab, Harman Research, Harman International, 30001 Cabot Dr, Novi, MI 48377, USA; anastasiya.bakhchina@harman.com (A.B.); evgeny.burashnikov@harman.com (E.B.);

**Keywords:** eye tracking, eye gaze, driving, cognitive load, driving simulation

## Abstract

Driving is a cognitively demanding task engaging attentional effort and working memory resources, which increases cognitive load. The aim of this study was to evaluate the discriminative capabilities of an objective eye tracking method in comparison to a subjective self-report scale (the NASA–Task Load Index) in distinguishing cognitive load levels during driving. Participants (*N* = 685) performed highway and urban driving in a fixed-base driving simulator. The N-Back test was used as a secondary task to increase cognitive load. In line with previous studies, the NASA–Task Load Index was shown to be an accurate self-report tool in distinguishing conditions with higher and lower levels of cognitive load due to the additional N-Back task, with best average accuracy of 0.81 within the highway driving scenario. Eye gaze metrics worked best when differentiating between stages of highway and urban driving, with an average accuracy of 0.82. Eye gaze entropy measures were the best indicators for cognitive load dynamics, with average accuracy reaching 0.95 for gaze transition entropy in the urban vs. highway comparison. Eye gaze metrics showed significant correlations with the NASA–Task Load Index results in urban driving stages, but not in highway driving. The results demonstrate that eye gaze metrics can be used in combination with self-reports for developing algorithms of cognitive load evaluation and reliable driver state prediction in different road conditions.

## 1. Introduction

According to the World Health Organization, road traffic injuries are the leading cause of death for children and young adults across the globe [1]. Approximately 1.3 million people die each year in traffic accidents; therefore, 2021–2030 has been declared the “Decade of Action for Road Safety”. Multiple factors may affect road safety, but existing research consistently highlights that drivers’ performance, including inattention and distraction, constitutes the major cause of critical situations leading to traffic accidents [2]. Psychophysiological and cognitive states are thought to be important contributors to drivers’ performance and judgement [3]. Technologies for continuous driver state monitoring with the aim to predict performance would contribute to promoting the optimal driver state (by analogy with the Yerkes–Dodson law [4]) and improving road safety.

Cognitive load (CL) is one of the most common aspects moderating drivers’ states and performance; therefore, the research into the dynamics of CL in the context of driving has gained particular interest in the automotive industry [2], aiming to advance the systems for automatic detection of drivers’ states and in-car intervention procedures. There are multiple ways to evaluate personal CL levels, and there is not a commonly accepted gold standard for validating newly developed diagnostic algorithms [5]. Wang and colleagues [6] described four types of metrics based on their sensitivity to CL and identified some modulating factors that may impact this sensitivity. Self-report and eye gaze parameters are among the most popular measures of drivers’ CL. Thus, the focus of this work was to evaluate two well-established subjective and objective methods measuring CL while driving on a large and diverse sample, in order to outline their benefits and limitations in various driving contexts. We hypothesized that combining subjective self-reports and objective eye gaze metrics could allow for new opportunities in the development of practical applications in the field of drivers’ state detection. Thus, in this study, we build on prior work by analysing subjective and eye gaze measures of CL on a large and demographically diverse sample of drivers. We directly compare the discriminative capabilities of self-report and eye gaze metrics within the same simulated highway and urban driving paradigm and evaluate the coherence of different measures of CL to determine what can serve best as a context-dependent ground truth for training driver state models.

CL can be defined as the attention control effort required to complete a task, which is limited by a working memory capacity and the cost of switching between tasks [7]. In line with this definition, CL is usually manipulated in experiments by applying a set of tasks contrasting lower and higher CL conditions [8,9,10]. In this study, we modulated CL levels using the N-Back task [9] because it is suitable for implementation in car driving simulations in an audial format and because it corresponds to the formal definitions of impaired or distracted driving, including the “phone-use” distraction scenario in the Euro NCAP protocol (Implementation 2026, Version 10.0, June 2025) [11]. Additionally, driving-related demand was manipulated by the road environment complexity (simple highway vs. urban roads with traffic).

Previous studies have compared CL evaluation approaches based on self-reports and physiological indicators [12,13,14,15]. However, it is difficult to make conclusions on the discriminative capabilities of different measures of CL from such studies because they have largely used different experimental protocols for manipulating CL, have used different measurements of CL on relatively small samples with individual biases, or have considered only one type of road environment for driving. In this study, we have sought to address these limitations by collecting data on a large sample, which would also allow us to control the factors of gender, age, and ethnic diversity. Most of the studies use self-report surveys to establish respondents’ experiences relevant to tasks performance [16]. Although self-report has its benefits (lower costs, easy to use, etc.), the reliability and validity of self-report as a measure of CL is still unclear and can vary across different types of tasks and conditions. Participants tend to rationalize their answers and give socially desirable responses, while individual differences often skew subjective ratings [17,18,19]. Although self-reports allow within-subject comparisons, possible individual differences in self-reported scores make it harder to compare them across experiments [17].

A significant limitation of self-report as a measure of CL is that it may affect primary task performance [18]. Therefore, self-reports cannot be collected with high temporal resolution (e.g., every 30 s or higher) and are primarily used to characterize a driver’s state within longer periods (e.g., 5 min). For some conditions, 5 min assessment intervals may be sufficient, but for driving, where external conditions and tasks change quickly, it is desirable to assess CL on shorter windows of analysis. A commonly used tool for validating a newly developed measure of CL is the NASA–Task Load Index (NASA TLX) survey [15,19,20]. Of particular importance is its successful implementation in the studies of cognitive distraction during car driving [15,21].

A wide range of physiological measures have been used in previous studies for estimating CL [22]: brain activity (EEG, fNIRS, fMRI), ocular metrics (blinks, pupil diameter), heart rate variability, and respiratory and endodermal signals (temperature, galvanic skin response) [15,23,24,25,26,27]. However, physiological measures also hold some limitations, e.g., their more expensive implementation, the way in which some of the available tools still cause interruptions in task performance, and the way in which their reliability in terms of distinguishing drivers’ states is limited by capabilities of recording sensors.

In this study, ocular motion measures have been selected as a physiological indicator of changes in a driver’s state, because this method can be applied non-intrusively, without interrupting behaviour. Ocular activity is highly relevant to driving behaviour. As visual modality is highly involved in the organisation of any human behaviour, providing the most differentiated, detailed, and complex interactions with the environment [28], eye movement and gaze parameters are expected to be sensitive to task complexity and could reflect changes in performance under various conditions. This idea is supported by conclusions of the extensive review by Ayres et al. [29], who found that eye gaze measures were the most sensitive to changes in CL levels, in comparison with other physiological measures. Table 1 lists eye gaze metrics that reflect CL levels most effectively, according to other published studies. Based on the results of these literature analyses, we selected the following eye gaze measures to include in our study: fixation duration, saccade velocity, number of blinks, gaze transition entropy (GTE), and stationary gaze entropy (SGE).

Fixation duration is an indicator of the level of challenge involved in interaction with visual information and the extent to which a person finds the visual input engaging. In the context of driving, fixation duration can provide insights into the difficulty of capturing and processing traffic information and the driver’s level of interest in what is within their field of vision [15]. Saccades are critical for navigating within the visual world, including driving. It is shown that saccadic velocity correlates with variations in sympathetic nervous system activation and, therefore, reflects changes in cognitive demand and emotional arousal [39]. Increased cognitive demand and distractions during driving have been shown to be associated with decreased saccade velocity [40,41,42]. Blink rate can be affected by external and internal factors, such as perceptual demand of tasks and cognitive demand. An auditory N-Back task during driving has been shown to be associated with an increased blink rate [43]. SGE reflects the global complexity of individual eye movement behaviour and overall gaze dispersion. The entropy measure of global complexity can better distinguish between baseline driving and conditions of high visual–spatial task load [44]. GTE is mostly considered as a measure of visual scanning efficiency [44,45], which is also relevant to driving, and it estimates the complexity of gaze transition patterns.

Previous work in aviation and driving has shown that both the spatial distribution and temporal organization of fixations are systematically related to CL and environmental complexity. Early studies of pilot scanning have reported that increases in task demand and display complexity are accompanied by changes in the breadth and regularity of visual scanning [46,47]. In the driving domain, it has been demonstrated that the spatiotemporal distribution of fixations is sensitive to drivers’ mental workload [48], while indices derived from the spatial distribution of fixations and entropy-based measures can distinguish different types and levels of task demand [49]. These findings suggest that entropy-like metrics are well suited to capture how drivers adapt their gaze behaviour when the visual environment becomes more complex or when additional cognitive tasks are imposed.

Several factors are potential limitations of eye-tracking technology in its application in driver state measuring. Individual differences, such as eye shape, eye makeup, tears etc., can disrupt the detection of eye features. Therefore, it is important to keep the gender balance and ethnic diversity in datasets used for developing state detection algorithms.

In this study, we analysed selected eye gaze metrics and self-reports collected from 685 drivers in a simulated driving task (highway and urban driving). We tested the discriminative capabilities and coherence of these measures of CL. We hypothesized that subjective and eye gaze measures may be more sensitive to different types and levels of CL, and that their discriminative capabilities may vary under different conditions (for example, Wang and colleagues [6] defined four types of metrics based on their sensitivity to CL). Thus, the goal of the study was to examine and compare the discriminative capabilities and coherence of self-report and eye gaze measures for CL estimation during driving in urban and highway environments, with and without an additional task. From the perspective of cognitive load theory [50], the present design separates two conceptually distinct sources of demand. The complexity of the road environment (urban vs. highway) primarily induces extraneous load, by increasing the amount and variability of task-irrelevant or context-driven information that the driver must monitor. In contrast, the additional task primarily induces intrinsic load, by adding working memory and decision-making demands that are intrinsic to the secondary task sequence but not directly related to basic vehicle control. Our 2 × 2 within-subject design orthogonally manipulates these two sources of load, allowing us to examine how self-report and eye-tracking measures respond to changes in extraneous and intrinsic load during driving.

Based on the above, we addressed the following research questions (RQs):

- RQ1. How do different types and levels of CL induced by road environment complexity and a secondary task modulate eye gaze dynamics and self-report measures during simulated driving?
- RQ2. To what extent do eye gaze and self-report measures differ in their ability to discriminate between lower and higher CL across typical driving conditions?
- RQ3. Under which driving conditions do eye gaze and self-report measures show the highest coherence, and can this coherence be used to identify context-dependent ground truths for CL detection?

## 2. Materials and Methods

### 2.1. Participants

All subjects volunteered to participate in this study and signed an informed consent. Data were collected from 868 participants. A final sample of 685 (54% male; age from 18 to 80 years old: Med = 37, M = 39, SD = 15.64) participants was selected for analysis. The inclusion criteria were as follows: (1) sufficient quality of eye gaze recordings and (2) adequate N-Back task performance. The selected participants identified themselves as being of African, Asian, European, Hispanic or Indian ethnic groups and reported their driving experience—from 1 to 45 years. More detail on the distributions of participants across ethnic groups and years of driving experience can be found in Table S1 and Table S2, respectively. They reported that at least one year had passed since they acquired their driving license and that they usually drive regularly, at least once a week. All participants reported that they were healthy, neurologically normal, not currently taking any psychoactive medication, and with normal-to-corrected vision, including no colour blindness.

All the participants were paid for taking part in the study and signed an informed consent. The study complied with the tenets of the Declaration of Helsinki and was approved by the National Center for Bioethics of the Scientific Psychology Center of Yerevan State University (2 June 2023, No. 23/05/01). All methods used in the study were performed in accordance with the relevant guidelines and regulations.

### 2.2. Driving Simulation

The experiment was performed in a fixed-base driving simulator developed using BeamNG.py library (BeamNG.tech). It included conventional in-vehicle equipment: a driver’s seat, steering wheel, and pedals (accelerator and brake, as in automatic transmission). A computer with high processing capability was synchronised with the simulator to record participants’ steering activity and vehicle location on the x, y, and *z* axes. The simulator generated images on three LED monitors located in a 180° semicircle around the participant at the distance of approximately 1120 mm (for more details, please, see Figure S1). Temperature and lighting were controlled during the experiment (19–21 °C, 80–100 lux). The in-vehicle acoustic environment was also appropriately simulated using standard BeamNG sound effects.

### 2.3. Experimental Tasks

Among other factors, driving performance is moderated by (1) road environment complexity (extraneous load) and (2) any additional tasks an individual is engaged in during driving that are unrelated to controlling and directing the vehicle (intrinsic load). These two factors were manipulated in the experiment to control the amount of extraneous and intrinsic CL administered in each condition.

**Figure 1:**
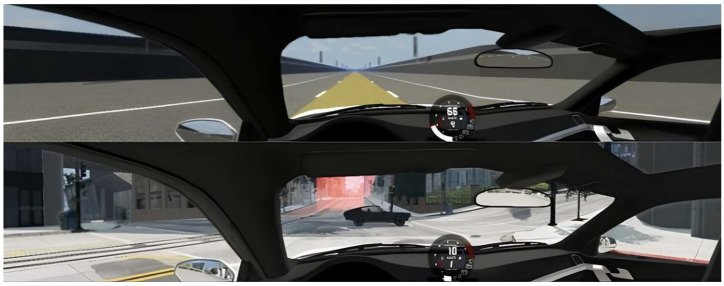
Screen view of the simulator environments. Highway driving scenario (**top**) and urban driving scenario (**bottom**).

The simulator was equipped for two types of driving scenarios: highway and urban. The highway scenario was considered a simple driving condition (lower CL), and the urban driving scenario was used as a complex driving condition (higher CL) (Figure 1). In the simple highway scenario, the road environment was flat, with no traffic or other disturbances. Participants were instructed to drive the vehicle respecting the speed limit and traffic rules. In the urban scenario, a standard city environment with traffic was simulated. Participants were instructed to drive the vehicle along the route indicated by red markings on the road while respecting the speed limit and traffic rules. There was no feedback on driving errors. The speed limit in both scenarios was 45 mph (72 km/h). In cases of accidental damage to the simulated vehicle, it was forcibly stopped and was reinitiated from the most recent checkpoint, from which the participant would continue driving.

### 2.4. N-Back Task

The N-Back task is clearly quantified way to create CL [6]. Therefore, the N-Back task was used as a secondary task to increase participants’ CL level during driving. We implemented a modified version of the auditory–verbal N-Back task, which is widely used in driving research and has validated in terms of its ability to impose varied levels of CL on drivers [51]. In each cycle of the N-Back task, participants listened to a pre-recorded series of 10 letters, separated by approximately 2.5 s intervals.

There were three levels of the N-Back task within each session: in 1-back, participants were asked to press the button (built into the steering wheel) each time two identical letters appeared back-to-back (e.g., OO); in 2-back, participants were asked to press the button each time two identical letters appeared in pairs separated by one letter in between (e.g., OEO); and in 3-back, participants were asked to press the button each time two identical letters appeared in pairs separated by two letters in between (e.g., OEAO).

In a 5 min test driving session, 1-, 2- and 3-back tasks were combined sequentially: first, 1-back and 2-back were presented each 3 times, and, finally, 3-back was presented twice, in the following order: 1-back, 2-back, 3-back, 2-back, 1-back, 2-back, 3-back, 1-back. An automated announcer repeated the short instruction to the participant before each task.

The scheme of N-Back task sequence in driving stages is presented in Figure S2. The difficulty of the N-Back task was varied within each session in order to model naturalistic conditions in which the level of CL fluctuates and is not maintained at the same level for the entire duration of the task. With a view to use these data for the future development of algorithms and models for the estimation of CL levels, we varied task difficulty to avoid overfitting such solutions to a constant level of CL. In all experiments selected for analyses, subjects showed acceptable N-Back task performance (percentage of correct answers >50%). Distributions of N-Back errors for experiments selected for analyses are given in Table S3.

### 2.5. Cognitive Load Questionnaire

Subjective CL ratings were collected using an adjusted NASA–Task Load Index (NASA TLX) survey [19,20], which is a commonly used tool for validating newly developed measures of CL. Of particular importance is its successful implementation in the studies of cognitive distraction during car driving [15,21]. After completing each of the experimental stages, participants responded to six questions on a nine-point Likert scale, ranging from “very low” (−4) to “very high” (+4). The survey was presented on a tablet screen mounted next to the driver’s seat. The following questions within the NASA TLX were used:

(a) How mentally demanding was the driving? (Mental demand scale)
(b) How physically demanding was the driving? (Physical demand scale)
(c) How hurried or rushed was the decision making during the driving? (Temporal demand scale)
(d) How successful were you in accomplishing the driving? (Performance scale)
(e) How hard did you have to work to accomplish your level of performance of the driving? (Effort scale)
(f) How stressed, irritated, and annoyed did you feel while performing the driving? (Frustration scale)

Additionally, the averaged answer (with an inversion of performance scale values) was calculated for each presentation of NASA TLX.

### 2.6. Experimental Procedure

To minimize the impact of circadian rhythms, all experiments were performed between 8 am and 6 pm. Subjects voluntarily chose a session that suited them best within 2 h time slots available in the schedule.

Prior to the experiments, participants were asked to ensure that they adhered to the following guidelines: have a normal amount of sleep the night before the experiment, do not have food or drinks containing caffeine for at least 2 h prior to the experiment, do not take any medications causing drowsiness for at least 8 h before the experiment, do not consume any alcohol for at least 24 h prior to the experiment, and do not smoke or engage in vigorous physical activities for at least 2 h prior to the experiment.

At the beginning of each experiment, the participants were asked to complete questionnaires gathering information on the quality of their last night’s sleep, caffeine and alcohol consumption, and the taking of any medication that could affect driving performance. They also responded to NASA TLX before driving. Then, participants were asked to sit down in the driving simulator. At the beginning of the experiment, participants were given an opportunity to familiarise themselves with the simulator environment by completing a 5 min training task. The main experiment contained four 5 min stages:

1. Simulated urban driving (UrbanDriving);
2. Simulated highway driving (HighwayDriving);
3. Simulated urban driving with simultaneous N-Back task (UrbanNback);
4. Simulated highway driving with simultaneous N-Back task (HighwayNback).

The order of the stages with driving was randomised for each participant to avoid order-induced bias. The stages were not repeated in the experiment. The total distance travelled within the experiment was ~22 km long.

### 2.7. Eye Gaze Recording and Analysis

Eye gaze was recorded using a commercial eye gaze tracker, Smart Eye (https://smarteye.se/, accessed on 17 October 2025). The principal scheme of eye gaze data collection in the driving simulation is presented in Figure S1. Two cameras for gaze tracking were installed within a horizontal black bar attached to the bottom of the central screen, above the steering wheel. The Smart Eye sampling rate was 60 Hz. Before each experiment, the eye tracking system was geometrically calibrated using the standard Smart Eye chessboard procedure to align the cameras and define the 3D tracking volume. Participants also completed a standard multi-point gaze calibration prior to driving in the simulator. Calibration was repeated when necessary if the experimenter observed clear misalignment between estimated gaze position and visible screen or road features. According to the manufacturer’s specifications, under recommended conditions, typical gaze accuracy is within an approximate 0.5–1.0° of visual angle, with comparable precision during stable fixations. Data were exported to custom-made Python 3.12.3 routines for subsequent analysis of saccades, fixations, and blinks.

Only data collected during driving stages were included in the computation of eye gaze metrics. We used the internal quality parameters of the eye gaze tracking system to filter out invalid data. For each frame, the Smart Eye system outputs several data quality variables, including head position quality and gaze direction quality, each ranging from 0 (tracking failed) to 1 (optimal tracking) and reflecting the number of cameras contributing to the estimate and the strength of iris detection, respectively. Frames with low head position quality or gaze direction quality below a set threshold (<1.0), or flagged as invalid (e.g., tracking loss), were discarded. Only frames classified as valid were included.

The points of intersection of the gaze direction vector with a mobile reference plane were determined. For each frame, a distinct reference plane was constructed based on the head direction vector at a fixed distance. The configuration of the plane was based on the equation of the plane through the normal vector to the plane and a point, as follows:

$$A\left(x-x_{0}\right)+B\left(y-y_{0}\right)+C\left(z-z_{0}\right)=0$$

where $\left(x_{0},\;y_{0},\;z_{0}\right)$ is the point of the gaze plane and $\left(A,B,C\right)$ is the normal vector.

The normal vector was derived from the head direction vector obtained from the head rotation matrix. The point of the gaze plane is a point plotted along the direction vector of the head from the position of the head to a given distance (in our case, the distance was 112 cm). Next, we determined intersections of the gaze direction vector with the reference plane. To achieve this, we used Gaussian elimination to solve a system of linear equations, as follows:

$$\left[\begin{array}{ccc} gaze\_direction[1] & gaze\_direction[0] & 0\\ 0 & gaze\_direction[2] & -gaze\_direction[1]\\ A & B & C \end{array}\right]\left[\begin{array}{c} x\\ y\\ z \end{array}\right]\;\;\;\;\;\;\;\;\;\;\;\;\;\;\;\;\;\;\;\;=\left[\begin{array}{c} gaze\_direction[1]*\;gaze\_position[0]-gaze\_direction[0]*\;gaze\_position[1]\\ gaze\_direction[2]*\;gaze\_position[1]-gaze\_direction[1]*\;gaze\_position[2]\\ D \end{array}\right]$$

In this approach, the matrix does not have a solution using the Gaussian elimination method in only two cases: when the line lies in the plane and when they are parallel. Based on the points of intersection of the eye direction vectors with the mobile projection plane, the main events of the eye movement are then calculated. Fixation duration, saccade velocity, and blink number were computed as in [52]. At 60 Hz sampling, fixations were defined as periods, during which the change in gaze direction between successive samples was <1° for at least 6 consecutive frames (≥100 ms), with an upper duration limit of 1200 frames (20 s). Saccades were defined as periods with inter-sample gaze direction changes ≥1°, durations between 2 and 12 frames (≈30–200 ms), and total amplitude ≤60°, and were further filtered using a velocity-dependent threshold and an amplitude-to-peak-velocity ratio (AVR) ≤10 to reject implausible events. Blinks were identified from the eyelid-opening signal using derivative and duration thresholds (5–180 frames, ≈83–3000 ms). All parameters were kept constant across participants and conditions. 

SGE and GTE were computed as in [45]. To calculate SGE and GTE, fixation coordinates were discretised by organising them into spatial bins of 30 × 30 pixels, which allowed for the generation of state spaces across the visual field with sufficient transition distributions. This binning approach follows previous work on gaze entropy in naturalistic driving tasks [45]. SGE quantifies how fixations are distributed across the visual scene, with higher values indicating a more dispersed allocation of gaze across different regions of interest. GTE captures the predictability of the gaze sequence by indexing the entropy of transitions between regions of interest; higher GTE reflects more variable, less stereotyped scan paths. Conceptually, SGE reflects the spatial dispersion of gaze, whereas GTE reflects the temporal organization of the scan path [45].

The metrics were calculated for eye gaze trajectory in overlapping 30 s windows with a 1 s step. This approach was applied with a view to the development of solutions for continuous monitoring, and it allows one to effectively control eye gaze data quality during analyses. Only frames classified as valid contributed to each 30 s window and only windows with a quality of raw gaze data higher than 50% were selected for the analysis. The metric values for selected windows were averaged for 300 s stages of driving. For each participant, we calculated the proportion of low-quality windows across the drive. Participants were retained in the analyses only if no more than 35% of their windows were low quality.

### 2.8. Data Analysis

We performed comparisons of NASA-TLX answers and eye gaze metrics between driving stages. Statistical analyses were performed using open-sourced python SciPy library. Distributions of variables were tested for normality using the Shapiro–Wilk test. We used an alpha level of 0.05 for all statistical tests.

To address RQ1, we conducted omnibus repeated-measures ANOVAs (RM-ANOVAs) and multivariate analyses of variance (MANOVAs) to examine the overall effects of driving stage on eye-movement behaviour and self-report measures. Driving stage (four levels: HighwayDriving, UrbanDriving, HighwayNback, and UrbanNback) served as a within-subject factor. Assumption checks indicated deviations from normality and equality of variances in the considered variables; therefore, Games–Howell post-hoc tests were used for pairwise comparisons between stages.

For eye-movement data, separate RM-ANOVAs were run for blink rate, saccade velocity, fixation duration, SGE, and GTE, complemented by a MANOVA including these five metrics as dependent variables. Separate RM-ANOVAs were conducted for each NASA-TLX dimension (mental demand, physical demand, temporal demand, performance, effort, and frustration), and a MANOVA was run with the six dimensions as dependent variables.

RQ2 was addressed using a discriminative accuracy analysis. As described above, eye gaze metrics were calculated within rolling 30 s windows with a 1 s step through each stage. Then they were averaged for each stage, and the averaged values were compared between the stages within the three pairs: HighwayDriving vs. UrbanDriving, HighwayDriving vs. HighwayNback, UrbanDriving vs. UrbanNback. These pairs of driving stages differed in either road environment complexity or the presence/absence of the secondary task. The levels of CL were modelled in the driving task as follows: (1) CL level during urban driving is higher than highway driving (UrbanDriving > HighwayDriving) and (2) CL level is higher in stages of driving with simultaneous N-Back task performance, compared with driving without an additional task (i.e., UrbanNback > UrbanDriving, and HighwayNback > HighwayDriving). Comparisons between urban and highway stages primarily index changes in extraneous load due to road environment complexity, whereas comparisons between stages of driving with the N-back and driving without any additional tasks within the same road type primarily index changes in intrinsic load due to the secondary task. This organisation allowed us to directly quantify the discriminative performance of each measure for these two conceptually distinct sources of CL. By contrasting NASA-TLX scale values and eye gaze metrics in pairs of stages with lower and higher CL (HighwayDriving vs. UrbanDriving, HighwayDriving vs. HighwayNback, and UrbanDriving vs. UrbanNback) we calculated their accuracy, in terms of distinguishing CL levels, as the percentage of subjects for whom the measurements obtained during stages reflected the expected differences between these stages [53].

**Figure 2:**
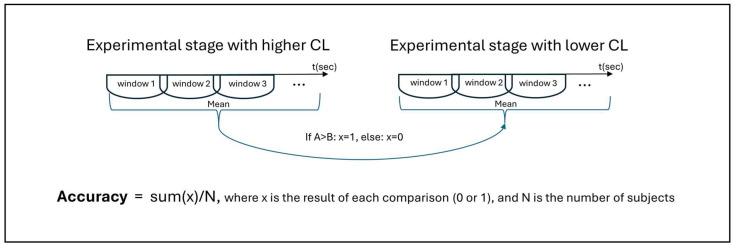
Accuracy calculation. Eye gaze metric values were calculated for each window of analysis and then values for all windows with acceptable data quality were averaged for each participant within each driving stage. An averaged value within one driving stage was compared with an averaged value for the stage of comparison within subjects. If an absolute difference between the values fit the expected dynamics, this comparison counted as 1, otherwise 0. Accuracy was calculated as the number of subjects with expected difference between the stages divided by the total number of subjects.

As shown in Figure 2, eye gaze metrics were calculated for each window of analysis and then values for all windows with acceptable data quality were averaged for each participant within each driving stage. An averaged value within one driving stage was compared with an averaged value for the stage of comparison within subjects. If an absolute difference between the values fit the expected dynamics, this comparison counted as 1, otherwise 0. The dynamics that were to be expected had been established based on the literature and were supported by our own results of the within-subject comparisons (see above). NASA-TLX scale answers were compared as one value for each stage of the pair. Thus, accuracy was calculated as the number of subjects with expected difference between the stages divided by the total number of subjects. Accuracy values could be in the range between 0 and 1, with values above 0.5 and closer to 1 indicating higher accuracy. This accuracy analysis provides an intuitive descriptive indicator of how consistently a given metric reflects the expected ordering of conditions and is particularly relevant for assessing the suitability of candidate measures for driver state monitoring.

To address RQ3, Spearman’s rank correlation coefficient was computed for analysis of relationships between NASA TLX answers and eye gaze indexes. We tested hypotheses about linear associations between subjective and physiological CL measures in different conditions, separately within each stage of driving. Interpretation of strength of correlation coefficients used in the article was based on the guide [54], where correlations with coefficients lower than 0.3 were considered as weak, 0.3–0.7 as moderate, and higher than 0.7 as strong.

To assess consistency of NASA-TLX scales and eye gaze metrics in CL evaluation, we calculated coherence between the metrics and stages of the experiment, using Lin’s concordance correlation coefficient, which tests how well bivariate pairs of observations conform relative to a gold standard [55], which, in our case, is present within the experimental stages. The experimental stages within each pair (as above, UrbanDriving > HighwayDriving, UrbanNback > UrbanDriving, and HighwayNback > HighwayDriving) were coded on a rank scale reflecting their CL level (i.e., 0 and 1). Coherence was calculated as Lin’s correlation between (1) the stages and eye gaze metrics (separately for each of the eye gaze metrics with the stage ranks, and then absolute values of obtained correlations were averaged), (2) the stages and NASA-TLX scales values (separately for each of the scales with the stage ranks and then absolute values of obtained correlations were averaged), and (3) between eye gaze metrics and NASA-TLX scales values (calculated for each eye gaze metric with each scale and then absolute values of obtained correlations were averaged). Absolute coherence values were then aggregated (mean, min, max) for the whole sample, with only significant correlations being used.

## 3. Results

### 3.1. Eye Gaze Metrics and NASA TLX Responses Across Different Driving Conditions

First, we addressed RQ1 and tested how different types and levels of CL induced by road environment complexity (highway vs. urban) and a secondary task modulate eye-gaze dynamics and self-report measures during simulated driving.

RM-ANOVAs on individual oculomotor metrics showed strong main effects of driving stage for all measures: blink rate, F(3, 2160) = 191.97, *p* < 0.001; saccade velocity, F(3, 2160) = 140.96, *p* < 0.001; fixation duration, F(3, 2160) = 191.90, *p* < 0.001; SGE, F(3, 2160) = 581.26, *p* < 0.001; and GTE, F(3, 2160) = 1361.96, *p* < 0.001. Games–Howell post-hoc comparisons (Table S4, also see Table S5 for descriptive statistics) revealed significant pairwise differences between driving stages for all measures. Specifically, blink rate, saccade velocity, fixation duration, and both entropy metrics differed significantly between most stage combinations, confirming that gaze dynamics were strongly modulated by the driving context.

To assess whether eye-movement behaviour differed globally across the four driving stages, we conducted a multivariate analysis of variance (MANOVA) with blink rate, saccade velocity, fixation duration, SGE, and GTE as dependent variables and driving stage as a within-subject factor. The multivariate test revealed a highly significant effect of driving stage on the combined set of eye-movement metrics: Hotelling–Lawley trace = 2.154, F(15, 5427.21) = 412.83, *p* < 0.001. These results demonstrate that the different driving stages produced profound changes in the multivariate profile of eye-movement behaviour, indicating systematic modulation of attentional allocation, gaze stability, scanning patterns, and visual (extraneous) cognitive load.

**Figure 3:**
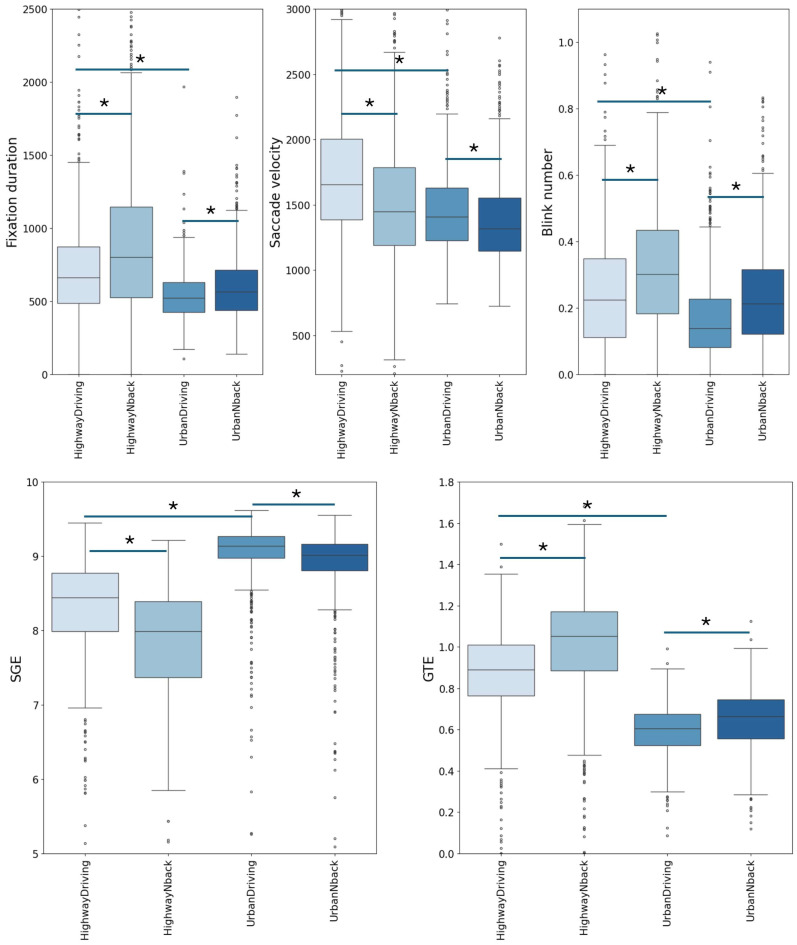
Distributions of eye gaze metrics. Median, quartiles, minimum, maximum and outliers are shown. Fixation duration (ms), saccade velocity (m/s), blink number, SGE, and GTE are shown for four stages of the experiment: HighwayDriving, HighwayNback, UrbanDriving, and UrbanNback; *—*p* < 0.001, Games–Howell post-hoc tests.

As can be seen from Figure 3, fixation duration and blink number were greater in highway driving, as compared with urban driving, and the addition of the N-Back task increased fixation duration and blinks in both scenarios. Thus, higher CL increases fixation duration and blinks, but urban driving is more dynamic than highway driving, which requires more frequent eye movements and less blinks, therefore the difference in external visual environment complexity was more prominent than internal CL differences for these parameters of eye gaze. Saccade velocity was lower in urban driving, as compared with highway driving, and this parameter decreased further with higher CL added by the N-Back task. Thus, this metric reflects CL dynamics related to the main task and any additional task in the same way—the higher the CL, the lower the saccade velocity. SGE was greatly higher for urban driving, compared with highway driving, but adding the N-Back task decreased SGE. GTE was higher in the highway condition, compared with urban driving, and the N-Back task affected this measure differently, as it decreased with additional CL in highway driving but increased with additional CL in urban driving.

**Figure 4:**
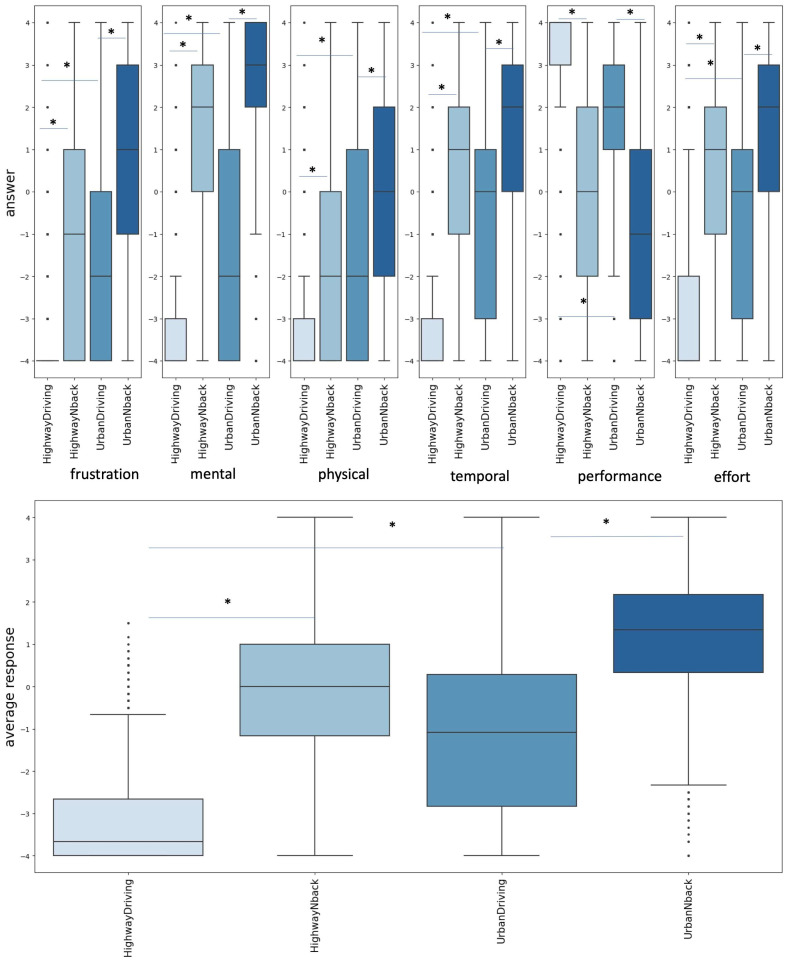
Distributions of NASA TLX responses. Median, quartiles, minimum, maximum and outliers are shown in four stages of the experiment—HighwayDriving, HighwayNback, UrbanDriving, UrbanNback—for each of the NASA TLX scales (**top**) and averaged response (**bottom**); *—*p* < 0.001, Games–Howell test.

To assess whether subjective CL varied across driving stages, we conducted separate RM-ANOVAs for each NASA-TLX dimension, with driving stage as a within-subject factor. All analyses revealed highly significant effects of driving stage, indicating that each CL component changed substantially over the course of the driving task. Significant effects were found for frustration, F(3, 2151) = 625.86, *p* < 0.001; mental demand, F(3, 2151) = 1432.65, *p* < 0.001; physical demand, F(3, 2151) = 377.19, *p* < 0.001; temporal demand, F(3, 2151) = 790.37, *p* < 0.001; performance, F(3, 2151) = 773.21, *p* < 0.001; and effort, F(3, 2151) = 698.92, *p* < 0.001. Pairwise Games–Howell comparisons revealed strong and systematic differences in NASA-TLX ratings between driving stages with different CL (Figure 4 and Table S6).

A MANOVA, including all six NASA-TLX dimensions as dependent variables and driving stage as the within-subject factor, corroborated these findings. The analysis revealed a robust and statistically significant multivariate effect of driving stage across all four test criteria: Hotelling–Lawley trace = 1.441, *p* < 0.001. The magnitude of these multivariate statistics reflects a large effect, indicating that the different driving stages elicited substantially distinct CL profiles.

As can be seen from Figure 4, NASA TLX ratings across all scales and the average value were greater in stages with higher CL, i.e., urban driving was rated as more demanding than highway driving, and driving with an additional N-Back task was rated as more demanding than driving without additional tasks. The maximal increase of NASA TLX ratings was observed for highway driving stages and was found to be lower in the urban driving stages comparison and lowest for urban vs. highway driving.

### 3.2. Discriminative Capabilities of Eye Gaze Metrics and NASA TLX Scales

Addressing RQ2, we examine to what extent do eye gaze and self-report measures differ in their ability to discriminate between lower and higher CL conditions across typical driving conditions.

To estimate how well eye gaze metrics distinguish CL levels, we calculated accuracy as the percentage of participants whose measures were different between the compared experimental stages in the expected direction, taking into account the results of sample distributions described above. Accuracy was calculated separately for fixation duration, saccade velocity, blink number, SGE, and GTE. For example, for SGE the expected dynamics is tested as follows: HighwayDriving > HighwayNback, UrbanDriving > HighwayDriving, UrbanDriving > UrbanNback. The eye gaze pattern depends on the external visual scene and cognitive state; therefore, it is expected that the discrimination between highway and urban driving is quite robust, the maximal accuracy was observed for GTE (0.95) and SGE (0.92). The least effective measures for differentiating urban driving and urban with N-Back task were fixation duration (0.65) and saccade velocity (0.64). Blink number discriminating rate was also consistent across all compared conditions (0.71–0.76). The details on the discriminating accuracy for each of the eye gaze metrics are shown in Table 2 and Figure S3.

Then, to estimate how well NASA TLX scales distinguished the CL levels modelled in the stages of the experiment, we calculated accuracy as the percentage of participants whose responses fit the expected dynamics, i.e., higher NASA TLX scores for stages with higher CL. Accuracy was calculated separately for all scales and their average answer (Table 3). For example, for the mental scale, the expected dynamics were as follows: HighwayDriving < HighwayNback, UrbanDriving < HighwayDriving, UrbanDriving < UrbanNback; or, for the following inverted performance scale: HighwayDriving > HighwayNback, UrbanDriving > HighwayDriving, UrbanDriving > UrbanNback. Physical scale was the least useful in discriminating conditions with different CL. The performance scale showed the best discriminative ability. In general, mental, temporal, performance, and effort scales as well as the averaged response showed good discriminative ability for conditions with different CL, especially so for HighwayDriving vs. HighwayNback. The lowest accuracy of NASA TLX in CL detection was observed for HighwayDriving vs. UrbanDriving. More details are given in Table 3 and Figure S4.

### 3.3. Correlation and Coherence Between Eye Tracking Metrics and NASA TLX Responses

Addressing RQ3, we performed a correlation and coherence analysis to test how well the dynamics of the eye tracking metrics corresponded to NASA TLX responses and under which driving conditions eye gaze and self-report measures show the highest coherence.

Significant correlations (Spearman rank correlation coefficient, see Table S8) between eye gaze metrics and responses to NASA TLX scales were observed for saccade velocity (r = −0.17 with averaged answer, r =−0.16 with performance scale, r = −0.13 with effort scale, r = −0.17 with temporal scale, r = −0.18 with mental scale, r = −0.17 with physical scale, r = −0.15 with frustration scale), GTE (r =−0.18 with physical scale, r =−0.19 with frustration scale, r =−0.13 with averaged answer), and blink number (r = 0.10 with mental scale, r = 0.10 with performance scale, r = 0.06 with effort scale). Then, we calculated Spearman rank coefficients between eye gaze metrics and answers to NASA TLX scales for each stage of the experiment, separately (Table S9), in order to test for any specific effects in different road environments and within different ranges of CL. Significant correlations were observed only for the stages of urban driving and urban driving with the N-Back task. In the UrbanDriving stage, correlations were observed for the following eye gaze metrics: blink number (r = 0.14 with averaged answer, r = 0.13 with effort scale, r = 0.17 with physical scale, r = 0.13 with frustration scale) and SGE (r = −0.14 with averaged answer, r = −0.13 with frustration scale, r = −0.15 with temporal scale, r = −0.16 with mental scale, r = −0.14 with physical scale). In UrbanNback stage, correlations were observed for the following eye gaze metrics: GTE (r = −0.18 with performance scale), SGE (r = −0.12 with averaged answer, r = −0.12 with effort scale, r = −0.15 with temporal scale), blink number (r = 0.12 with performance scale), and fixation duration (r = −0.14 with performance scale). These results show that eyes gaze measures and self-estimated evaluations of CL level correlate only in the urban environment and, one can guess, do not persist within the entire range of CL levels, only when CL is relatively high.

**Figure 5:**
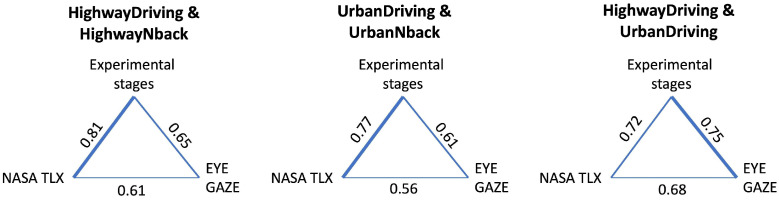
Coherence between stages, eye gaze metrics and NASA TLX. Mean Lin’s correlation values are shown for the HighwayDriving and HighwayNback, UrbanDriving and UrbanNback, and HighwayDriving and UrbanDriving stages. NASA TLX shows better coherence with the CL stages when differentiating driving with and without an additional task. Eye gaze metrics show better coherence with the stages differentiating driving in different road environments.

Finally, we analysed coherence between NASA-TLX answers, eye gaze metrics and experimental stages with different CL levels (see Figure 5, and Table S10). The coherence coefficient reflects the consistency in the dynamics of two metrics; therefore, when considering this coefficient between all metrics that relate to certain aspects of CL, we expected to find the most consistent one. As can be seen from Figure 5, NASA TLX shows better coherence with the CL stages when differentiating CL related to an additional task. Eye gaze metrics show better coherence differentiating between the highway and more complex urban environments.

## 4. Discussion

In this work, we aimed to explore how objective physiological measures of eye gaze could be used for estimating the dynamics of CL in a driving simulation task. We obtained and analysed an unusually large dataset for studies in psychophysiology (N = 685), which allowed us to achieve consistent average results representing the target population of drivers. The primary aim was to define conditions when the eye gaze metrics and NASA-TLX self-report scales successfully discriminate between contexts with higher and lower CL, and to verify under which conditions each of these measures performs better, with a view to use eye gaze and self-report tools in combination for the development of effective driver monitoring solutions.

We analysed NASA-TLX responses and eye gaze metrics in several conditions: driving in highway and urban environments, and with or without an additional cognitive task (the N-Back task) increasing CL. The overall results provide evidence that CL modelled in each of the driving stages had a large and systematic impact on both oculomotor behaviour and subjective ratings of CL. Strong CL effects were found for all individual gaze and self-report measures. Interpreted together with the pairwise accuracy analyses, these results indicate that changes in road environment complexity (extraneous load) and secondary task demands (intrinsic load) jointly reshape both the structure of gaze behaviour and the configuration of perceived workload, albeit with partially distinct sensitivities of the different measures.

In general, our study replicated previous results on CL estimated using a validated NASA-TLX questionnaire on drivers, but on a much bigger sample of participants [19]. The differences demonstrated between stages with higher and lower CL levels for both eye gaze metrics and self-report measures are in line with previous studies of CL (e.g., see [21,32]). Our large sample size allowed for an analysis of accuracy in distinguishing CL levels using each of the metrics in isolation on a diverse population of drivers. This analysis highlights the strengths and limitations of objective eye gaze measures and self-reported measures of CL, and supports the approach of combining the two types of measures in driver monitoring solutions.

Eye gaze metrics showed higher accuracy at discriminating between different road environments: highway vs. urban. These two driving environments differed in complexity, reflected in the number of elements relevant to the main driving task performance. Research shows that road environment landmarks and navigational demands moderate the CL levels experienced by drivers. A highway road environment is shown to have less stress-inducing objects and events, as compared with an urban road environment, and this is reflected in the drivers’ heart rates [53] and facial expressions [56]. Moreover, driving performance has been shown to be affected by road landscape complexity, with moderate complexity contributing to optimal performance [57]. In our study, the eye gaze metrics reflected the distinct patterns of eye movement specific to driving in each of the environments. During urban driving, fixations are shorter in duration and their spatial distribution is more varied, while the transition pattern is less complex with lower saccade velocity, and drivers blink less frequently than when driving on a highway. This eye gaze dynamic reflects the attentional demands specific to the urban environment: drivers need to be aware of the situation on a road which contains more elements and changes more frequently than on a highway. With the addition of a secondary task, fixations increase in length but cover less of the surrounding environment, with more chaotic transition patterns, reduced saccade velocity and increased blinking frequency. When on the highway, drivers tend to look forward with longer and less distributed fixations. The gaze transition pattern is less predictable on the highway, with higher saccade velocity indicating more random shifts of attention. When a secondary task is added, similar to urban driving, both fixation duration and blink frequency increase, an increase which is accompanied by decreases in saccade velocity and overall gaze dispersion and a more random transition pattern. These changes, overall, demonstrate a decreased attentional control when driving with an additional task, or distraction.

Among the eye gaze metrics, the estimates of gaze entropy, including SGE and GTE, showed the best accuracy in distinguishing conditions with higher and lower CL. This can be explained by their greater relevance to the visual spatial orientation and scan paths. GTE was the most discriminative metric for distinguishing urban from highway driving, with substantially higher values in the urban stages. This pattern suggests that drivers are engaged in more variable sequences of gaze shifts when navigating complex urban scenes, distributing their fixations across a broader set of task-relevant elements such as intersections, pedestrians, traffic lights, and parked vehicles. In contrast, highway driving elicited more stereotyped scan paths dominated by repetitions of a limited set of gaze shifts (e.g., far-road monitoring and periodic mirror checks), resulting in lower GTE. This interpretation is consistent with classic studies of pilot scanning, which reported that changes in task demand and display complexity are reflected in the structure of visual scanning patterns [46,47], and with more recent work showing that the spatiotemporal distribution of fixations provides a sensitive indicator of mental workload in both aviation and driving tasks [48,49].

Maggi and Di Nocera [49] further showed that different types of task demand can lead to either more dispersed or more clustered fixation patterns, with entropy measures tracking these changes over time. In our study, the N-Back task mainly increased CL on relatively simple highway scenes, whereas the urban environment increased visual and situational complexity. The strong discriminative performance of GTE for urban vs. highway stages aligns with the idea that entropy-based indices are particularly sensitive to changes in the structure and richness of the visual environment elevating extraneous load.

Shiferaw and colleagues [58] consider SGE and GTE within the model of gaze orientation. They define GTE as a measure of visual scanning efficiency that underlies the overall gaze dispersion measured by SGE. It is also hypothesized that GTE provides an estimation for the level of top-down modulation in gaze control, suggesting that there is a theoretically optimal GTE range for a given task, where the level of uncertainty is relative to the complexity of the visual environment and task requirements [59]. An increase in GTE may indicate stronger top-down modulation, but if it is increased above the optimum (e.g., in case of anxiety), it may provide interference to gaze behaviour. Decreased GTE, consequently, is associated with reduced top-down modulation and may also negatively affect performance. Depending on the visual requirement of the task, non-optimal changes in GTE can lead to an increase or reduction in overall spatial dispersion of gaze as measured through SGE, reflecting a mismatch between task requirement and overall gaze allocation. In our case, an additional task generated a distraction associated with an increased attentional demand and intrinsic load, which can be viewed as an increased top-down modulation reflected in the observed changes in GTE and SGE.

Thus, the dynamics of the eye gaze metrics reflected the characteristics of the external visual environment as well as internal cognitive efforts to perform the main and secondary tasks. Interestingly, with the addition of the N-Back task during urban driving, the dynamics of most of the metrics, including fixation duration, blink number, SGE and GTE, shifted towards the distributions observed in simpler highway driving. This can be interpreted as a decrease in the complexity of interactions between an individual and the external visual environment with rising internal cognitive efforts, which suggests that the perception of the external environment may be less detailed and reduced in such conditions. This is in line with other studies that tested gaze entropy in driving with an additional cognitive task [32,60].

Certain sets of eye gaze metrics in combination can potentially be used to detect changes in both types of CL [50]: extraneous, i.e., CL rising due to external visual environment complexity, and intrinsic, such as CL due to internal distraction with an additional task. This can be relevant for algorithms of CL detection, particularly distinguishing between different types of CL (e.g., intrinsic as opposed to extraneous [50]). The experimental paradigm implemented in this study creates opportunities for acquiring data that may be used for detecting the level of CL as well as its type. Although we only report five selected eye gaze metrics, it is important to note that recorded eye gaze data allow for the calculation of a much wider range of metrics, which could be useful in estimating CL. Future extensions of this study may include using other less conventional eye gaze metrics in addition to what we report here and apply machine learning tools to increase the accuracy and reliability in distinguishing CL levels during driving.

The NASA-TLX scale values exhibited high accuracies in differentiating between driving stages with and without an additional cognitive task. However, the accuracy of self-report measures, similar to eye gaze measures, was lower when differentiating urban driving conditions with and without the additional task. The lowest accuracy of self-report was observed when comparing urban and highway driving. This indicates that drivers are generally good at estimating perceived demand related to intrinsic load, especially when they compare a very simple condition (highway driving) and a condition of increased difficulty (highway driving with an additional task). When the main task is harder (urban driving), the same additional task is perceived as a smaller increase in CL, as measured by the NASA TLX scale. This potentially may create risks on roads because the drivers may underestimate an increased demand due to a secondary task in more complex road situations. The performance scale of NASA-TLX and the averaged response were the most accurate at distinguishing between different CL conditions, as compared with the other subscales. Interestingly, other studies have reported the effort scale to be the most effective in CL evaluation [19]. This may indicate that accuracy of NASA-TLX subscales depends on the type of task used for modulating CL and type of the main task.

In order to assess the relationship between subjective and objective estimates of CL within each of the conditions, we conducted a correlation analysis. We found that correlation between the NASA-TLX scales and eye tracking metrics was observed in the urban driving but not in the highway driving stages. We believe that this can be explained by the low dispersion and diversity of NASA-TLX scores between participants in the simple highway condition even as the distribution of eye tracking metrics had a higher between-subject variation. In the urban environment, both subjective responses and eye tracking metrics had sufficient variations, with a number of significant correlations observed between variables. Thus, in simple highway conditions with low variation in CL and its subjective assessment, it was easier to achieve higher discriminative accuracies. At the same time, it is much more challenging to discriminate between CL conditions in more complex urban driving with higher within- and between-subject variation in variable distributions. The correlations between self-reports and objective eye gaze metrics in such conditions suggest that these measures could be combined in order to achieve higher accuracies in CL discrimination. These results also demonstrate how important it is to validate methods of CL estimation in different road types and driving conditions.

The coherence analysis between NASA-TLX responses, eye gaze metrics, and stages with different levels of CL was aimed to identify the most consistent metrics in relation to CL. On average, none of the pairs of metrics demonstrates a consistency higher than 0.8. We found that the greatest average coherence was observed between driving stages and NASA-TLX responses in highway driving when compared with highway driving with an additional task, and between the driving stages and eye gaze metrics when comparing highway and urban driving. The lowest consistency between all three variables (experimental stages, self-reports and eye gaze measures) was observed in the urban environment. This demonstrates that the accurate detection of an increased CL level in a situation when it is already high is a more challenging task, one that requires special attention and possibly new approaches. Taken together, these results indicate that eye gaze metrics and self-reports could potentially be complimentary approaches in the development of reliable systems for CL monitoring, especially in complex conditions, such as detecting driver distraction in urban driving. For example, in addition to continuous state monitoring based on eye gaze (and other physiological parameters), self-report tools could be implemented in the form of a voice assistant powered by a conversational artificial intelligence (AI) model.

Selecting a valid and reliable ground truth is one of the most important steps in the development of diagnostic and predictive models of the human state. The ground truth serves as the “gold standard” observations describing the target state. Thus, the accuracy and reliability of the model depends on the accuracy and reliability of the ground truth. There are multiple approaches to the ground truth selection. For example, subjective evaluations are predicted based on eye gaze and other objective parameters and, vice versa, the dynamics of objective parameters are predicted based on subjective responses. Both such approaches are limited by the characteristics of the chosen reference, or benchmark. We suggest applying the coherence analysis to guide the selection of variables that are more suitable for the ground truth in different conditions. Thus, we consider the analysis of coherence as a new way to assess the effectiveness of a metric as a standard for a studied characteristic of the human state in different contexts. Potentially, this is an effective method of selecting the most consistent ground truth for artificial neural network training for the whole range of conditions.

Understanding the specific range of conditions in which different approaches in state detection exhibit their maximum effectiveness is crucial for optimizing the development of human–machine interfaces (HMIs), providing the computer with more detailed information about the subject. By identifying the strengths and limitations of physiological signal analysis and self-reporting in evaluating CL, our research contributes to the advancement of more accurate and reliable methods for assessing CL in real-world scenarios. Overall, the findings from our study have the potential to guide the future development of HMIs by providing insights into the most appropriate situations to employ physiological signal analysis and self-reports for assessing CL, not only in the context of driving but also in other applied fields.

### Limitations

The main limitation of this study, as we see, is the use of a driving simulation, without comparing the same variables in real driving. Previous studies have shown that real driving and simulated driving are comparable for measuring at least some driving performance indexes but in the simulated condition the effects of higher amplitude are usually expected [61,62,63]. It has been shown for sleepiness, cannabis smoking, alcohol intake and some other experimental conditions, that their effects can be studied in real and simulated environments, but that self-evaluation is always more affected in a simulated environment. In general, comparisons with real driving imply the relative external validity of simulators; however, results obtained in a driving simulator may need to be validated and calibrated against real driving in various conditions, which we plan to do in continuation of this research.

Among other potential limitations, our study did not specifically address the impact of such factors as participants’ individual differences, chronotypes, fatigue, familiarity with driving simulators, etc. We have taken measures to control some of these factors, as detailed in the Methods section, but future research is needed to incorporate any of their possible effects to enhance the accuracy and reliability of CL assessment.

In addition, driving stages in our experiments lasted only 5 min. This is a limitation that prevents us from making conclusions about prolonged driving with increased CL. We can hypothesize that prolonged driving with increased CL would facilitate conditions for overload and stress which would be reflected in eye gaze dynamics. Further research is needed to establish how well self-estimation and eye gaze metrics can differentiate increased CL conditions during prolonged driving.

## 5. Conclusions

In conclusion, the results of this study provide insights into the use of eye gaze metrics and NASA-TLX scales in estimating CL in simulated driving tasks. Our large dataset (N = 685) allowed for a comprehensive analysis with consistent average results representative of the target population of drivers. Both eye gaze metrics and self-report measures exhibited statistically significant differences between stages with higher and lower CL levels, reinforcing previous research. Furthermore, our findings highlight the specific contexts in which each measure is most effective: eye gaze metrics are particularly sensitive to the road environment and distinguish well between CL conditions related to road types while self-report is an accurate tool for capturing increases in CL due to a secondary task or distraction, particularly on simple highway roads. Combining self-reports and objective physiological markers of CL, such as eye gaze, could provide a more comprehensive understanding of CL and lead to the development of more accurate and reliable solutions for driver monitoring systems. Thus, our study contributes to the advancement of methods for assessing CL, offering potential guidance for the future development of practical applications in automotive and other industries.

## 6. Patents

Filimonov, A.V.; Filatova, A.S.; Burashnikov, E.P.; Shishanov, S.V.; Demareva, V.A.; Shishalov, I.S.; Devyatkin, A.S.; Sotnikov, M.S.; Burova, A.G.; Kilyazov, V.V.; et al. System and method for determining cognitive demand. International Patent Application WO 2022/055383 A1, 17 March 2022. https://patents.google.com/patent/WO2022055383A1/en accessed on 17 October 2025.

Bakhchina, A.V.; Shishalov, I.S.; Nevaykina, M.Y.; Yakimov, A.K.; Devyatkin, A.S.; Sotnikov, M.S.; Filimonov, A.V.; Borzikov, V.V. Driver psychophysiological state detection. U.S. Patent Application US 2024/0326830 A1, 3 October 2024. https://patents.google.com/patent/US20240326830A1/en accessed on 17 October 2025.

## Figures and Tables

**Table 1:** Examples of studies with significant eye gaze dynamics under different types of CL tasks.

Measures	Tasks	References
Fixation-based parameters	Search for errors in the text, flying simulation tasks, modified 2-back task	[15,25,30]
Saccade-based parameters	Search for errors in the text	[30]
Pupil dilation (for constant light conditions)	Driving under critical situations	[31,32]
Focus on relevant areas	Studying a map stimulus in a free-viewing condition	[33]
Average velocity of micro saccadic eye movement	Eye-gaze-controlled interfaces, instantaneous perception of developing road hazards, abstract figures operation	[34,35,36]
Blink-based parameters	Instantaneous perception of developing road hazards, multi-attribute decision making	[34,37]
Gaze entropy	Driving simulation, virtual simulations of laparoscopic exercises	[32,38]

**Table 2:** Accuracy calculated for eye gaze metrics.

Metrics	HighwayDriving vs. HighwayNback	UrbanDriving vs. UrbanNback	HighwayDriving vs. UrbanDriving
Blink number	0.76	0.76	0.71
Saccade velocity	0.71	0.64	0.75
Fixation duration	0.64	0.65	0.74
SGE	0.81	0.75	0.92
GTE	0.79	0.73	0.95
Average	0.74	0.71	0.82

**Table 3:** Accuracy calculated for NASA TLX scales.

Scales	HighwayDriving vs. HighwayNback	UrbanDriving vs. UrbanNback	HighwayDriving vs. UrbanDriving
Frustration	0.67	0.73	0.62
Mental	0.90	0.88	0.63
Physical	0.53	0.58	0.58
Temporal	0.83	0.70	0.72
Performance	0.96	0.92	0.91
Effort	0.83	0.70	0.69
Averaged response	0.95	0.91	0.87
Average	0.81	0.77	0.72

## Data Availability

The datasets presented in this article are not readily available because of ethical and legal restrictions. Requests to access the datasets should be directed to the corresponding author.

## Supplementary Materials

The following supporting information can be downloaded at https://www.mdpi.com/article/10.3390/jemr19010001/s1, Table S1. Demographic data: ethnic groups declared by participants. Table S2. Demographic data: years of active driving experience. Figure S1. Principal scheme of eye gaze data collection in the driving simulator. Figure S2. Principal scheme of the N-Back task sequence. Table S3. The N-Back task performance metrics. Table S4. Games–Howell post-hoc comparisons for eye gaze metrics. Table S5. Descriptive statistics for the eye gaze metrics in experimental stages and Wilcoxon test results. Table S6. Games–Howell post-hoc comparisons for NASA TLX scales. Figure S3. Percentages of subjects with expected difference in eye gaze metrics between stages with higher and lower CL. Table S7. Self-reported cognitive load levels in NASA TLX scales. Figure S4. Percentages of subjects with expected difference in NASA TLX scale values between each pair of stages with higher and lower CL. Table S8. Correlation between eye gaze and self-report measures across all experimental stages. Table S9. Correlation between eye gaze and self-report measures for each experimental stage separately. Table S10. Absolute coherence values between stages, eye gaze metrics and NASA TLX responses in pairs of stages with different levels of CL.

## Abbreviations

The following abbreviations are used in this manuscript:

CL	Cognitive load
NASA TLX	NASA–Task Load Index
NCAP	New Car Assessment Program
GTE	Gaze transition entropy
SGE	Stationary gaze entropy
AI	Artificial intelligence
HMI	Human–machine interface
