# Supplementary material for: Discriminative Capabilities of Eye Gaze Measures for Cognitive Load Evaluation in a Driving Simulation Task
Source: J Eye Mov Res. 2025 Dec 24;19(1):1. doi: 10.3390/jemr19010001 (PMC12821647; doi:10.3390/jemr19010001)
Supplement: Supplementary file 1 [file jemr-19-00001-s001.zip › jemr-3963226-supplementary.pdf]

# Supporting Information

## Table S1. Demographic data: ethnic groups declared by participants.

| Ethnic group | N |
|---|---|
| African | 40 |
| Asian | 94 |
| European | 394 |
| Hispani | 23 |
| Indian | 79 |
| Other group | 30 |
| Refuse to answer | 25 |

## Table S2. Demographic data: years of active driving experience.

| Years of active driving experience | N |
|---|---|
| <1 | 126 |
| >1 and <5 | 136 |
| >5 and <15 | 221 |
| >15 and <45 | 194 |
| >45 | 8 |

## Fig. S1. Principal scheme of eye gaze data collection in the driving simulator.

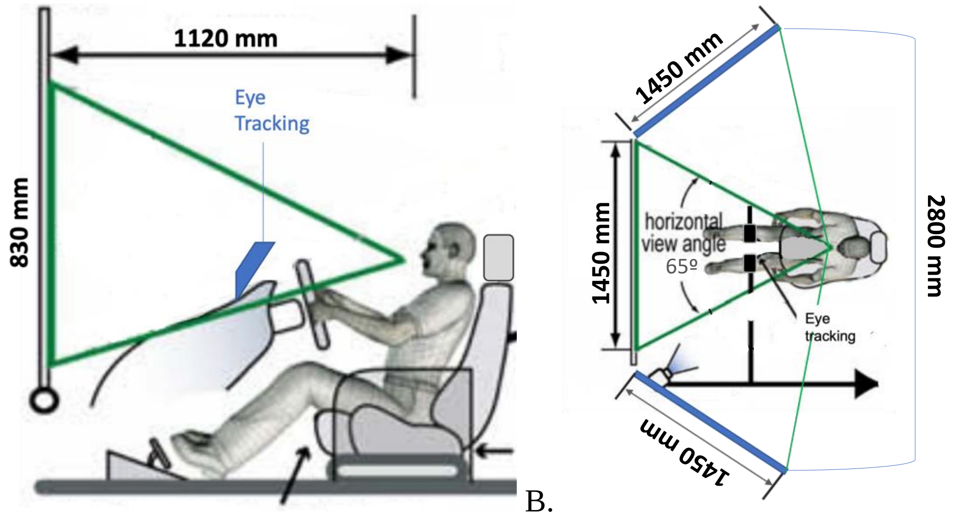


A. B.

**Fig. S2. Principal scheme of the N-Back task sequence.**

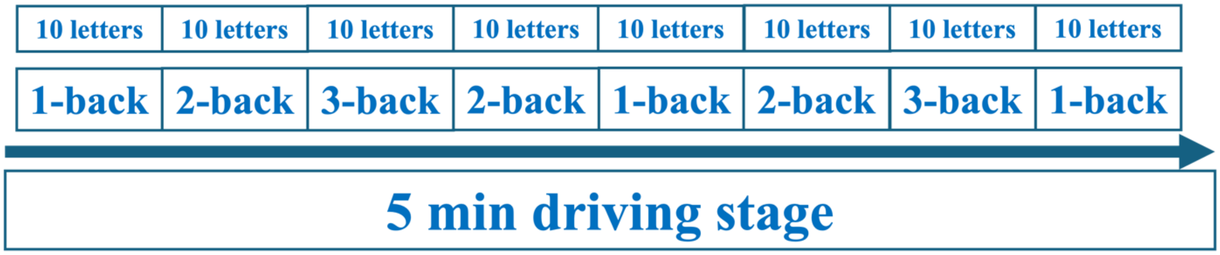


**Table S3. The N-Back task performance metrics**. Mean, standard deviation (std), median (50%), and quartile (25% and 75%) values are shown for false alarms and missed target stimulus events in 1-back, 2-back, and 3-back tasks.

| Stage | | false alarm 1-back | false alarm 2-back | false alarm 3-back | missed event 1-back | missed event 2-back | missed event 3-back | false alarm sum | missed event sum |
|---|---|---|---|---|---|---|---|---|---|
| Highway Nback | **mean** | 1.95 | 4.27 | 3.33 | 1.44 | 3.62 | 4.43 | 9.55 | 9.50 |
| | **std** | 3.02 | 3.30 | 2.13 | 2.61 | 2.76 | 2.15 | 7.36 | 6.30 |
| | **25%** | 0 | 2 | 2 | 0 | 2 | 3 | 5 | 6 |
| | **50%** | 1 | 4 | 3 | 0 | 3 | 4 | 8 | 8 |
| | **75%** | 2 | 6 | 4 | 1.75 | 5 | 6 | 13 | 12 |
| Urban Nback | **mean** | 2.97 | 4.00 | 2.50 | 4.10 | 6.09 | 5.12 | 9.48 | 15.31 |
| | **std** | 2.77 | 2.91 | 2.10 | 3.04 | 2.97 | 2.08 | 6.63 | 6.91 |
| | **25%** | 1 | 2 | 1 | 2 | 4 | 4 | 5 | 10 |
| | **50%** | 2 | 4 | 2 | 4 | 6 | 5 | 9 | 15 |
| | **75%** | 4 | 6 | 4 | 6 | 8 | 6 | 13 | 19 |

**Table S4. Games–Howell post-hoc comparisons for eye gaze metrics**

| Compared stages | Mean Difference | p-value | Effect Size (g) |
|---|---|---|---|
| **Blink Number** | | | |
| HighwayDriving – UrbanDriving | 0.07 | < .001 | 0.47 |
| UrbanDriving – UrbanNback | −0.06 | < .001 | −0.42 |
| HighwayDriving – HighwayNback | −0.08 | < .001 | −0.38 |
| **Saccade Velocity** | | | |
| HighwayDriving – UrbanDriving | 258.48 | < .001 | 0.59 |
| UrbanDriving – UrbanNback | 75.37 | < .001 | 0.22 |
| HighwayDriving – HighwayNback | 197.77 | < .001 | 0.37 |

| | | | |
|---|---|---|---|
| **Fixation Duration** | | | |
| HighwayDriving – UrbanDriving | 198.64 | < .001 | 0.59 |
| UrbanDriving – UrbanNback | −64.22 | < .001 | −0.30 |
| HighwayDriving – HighwayNback | −185.63 | < .001 | −0.34 |
| **SGE** | | | |
| HighwayDriving – UrbanDriving | −0.81 | < .001 | −0.91 |
| UrbanDriving – UrbanNback | 0.15 | < .001 | 0.26 |
| HighwayDriving – HighwayNback | 0.49 | < .001 | 0.41 |
| **GTE** | | | |
| HighwayDriving – UrbanDriving | 0.27 | < .001 | 1.62 |
| UrbanDriving – UrbanNback | −0.05 | < .001 | −0.39 |
| HighwayDriving – HighwayNback | −0.13 | < .001 | −0.55 |

**Table S5. Descriptive statistics for the eye gaze metrics in experimental stages and Wilcoxon test results.** * - p<0.01 in comparison of HighwayDriving and HighwayNback stages. & - p<0.01 in comparison of UrbanDriving and UrbanNback stages. # - p<0.01 in comparison of UrbanDriving and UrbanNback stages.

| Eye gaze metrics | Percentiles and average values | Highway Driving | Highway Nback | Urban Driving | Urban Nback | Wilcoxon test | Paired t-test |
|---|---|---|---|---|---|---|---|
| Fixation Duration | 25% | 485.77 | 527.67 | 425.59 | 439.78 | * & # | * & # |
| | 50% | 663.40 | 802.74 | 524.22 | 566.18 | | |
| | 75% | 872.78 | 1144.72 | 630.72 | 715.59 | | |
| | mean | 735.86 | 921.49 | 537.22 | 601.44 | | |
| Saccade velocity | 25% | 1386.41 | 1189.64 | 1226.21 | 1145.07 | * & # | * & # |
| | 50% | 1653.57 | 1446.13 | 1406.82 | 1315.31 | | |
| | 75% | 2003.11 | 1784.06 | 1628.36 | 1552.89 | | |
| | mean | 1715.81 | 1518.04 | 1457.33 | 1381.96 | | |
| Blink number | 25% | 0.11 | 0.18 | 0.08 | 0.12 | * & # | * & # |
| | 50% | 0.22 | 0.31 | 0.14 | 0.21 | | |
| | 75% | 0.34 | 0.43 | 0.23 | 0.32 | | |
| | mean | 0.25 | 0.34 | 0.17 | 0.24 | | |
| SGE | 25% | 7.98 | 7.36 | 8.97 | 8.81 | * & # | * & # |
| | 50% | 8.44 | 7.98 | 9.13 | 9.02 | | |
| | 75% | 8.77 | 8.39 | 9.26 | 9.16 | | |
| | mean | 8.21 | 7.71 | 9.02 | 8.86 | | |
| GTE | 25% | 0.76 | 0.88 | 0.52 | 0.56 | * & # | * & # |
| | 50% | 0.89 | 1.05 | 0.61 | 0.66 | | |
| | 75% | 1.01 | 1.17 | 0.68 | 0.75 | | |
| | mean | 0.87 | 1.01 | 0.59 | 0.65 | | |

**Table S6. Games–Howell post-hoc comparisons for NASA TLX scales.**

| Compared stages | Mean Diff (A–B) | p-value | Effect Size (g) |
|---|---|---|---|
| **Frustration scale** | | | |
| HighwayDriving – UrbanDriving | −1.97 | < .001 | −0.98 |
| UrbanDriving – UrbanNback | −2.38 | < .001 | −0.92 |
| HighwayDriving – HighwayNback | −2.35 | < .001 | −1.18 |
| **Mental scale** | | | |
| HighwayDriving – UrbanDriving | −1.90 | < .001 | −0.91 |
| UrbanDriving – UrbanNback | −3.47 | < .001 | −1.60 |
| HighwayDriving – HighwayNback | −4.25 | < .001 | −2.15 |
| **Physical scale** | | | |
| HighwayDriving – UrbanDriving | −1.70 | < .001 | −0.80 |
| UrbanDriving – UrbanNback | −1.35 | < .001 | −0.53 |
| HighwayDriving – HighwayNback | −1.52 | < .001 | −0.74 |
| **Temporal scale** | | | |
| HighwayDriving – UrbanDriving | −2.29 | < .001 | −1.09 |
| UrbanDriving – UrbanNback | −1.97 | < .001 | −0.84 |
| HighwayDriving – HighwayNback | −3.28 | < .001 | −1.59 |
| **Performance scale** | | | |
| HighwayDriving – UrbanDriving | 1.49 | < .001 | 0.78 |
| UrbanDriving – UrbanNback | 2.54 | < .001 | 1.14 |
| HighwayDriving – HighwayNback | 2.93 | < .001 | 1.50 |
| **Effort scale** | | | |
| HighwayDriving – UrbanDriving | −2.08 | < .001 | −0.91 |
| UrbanDriving – UrbanNback | −1.98 | < .001 | −0.85 |
| HighwayDriving – HighwayNback | −3.31 | < .001 | −1.53 |

**Fig. S3. Percentages of subjects with expected difference in eye gaze metrics between stages with higher and lower CL.**

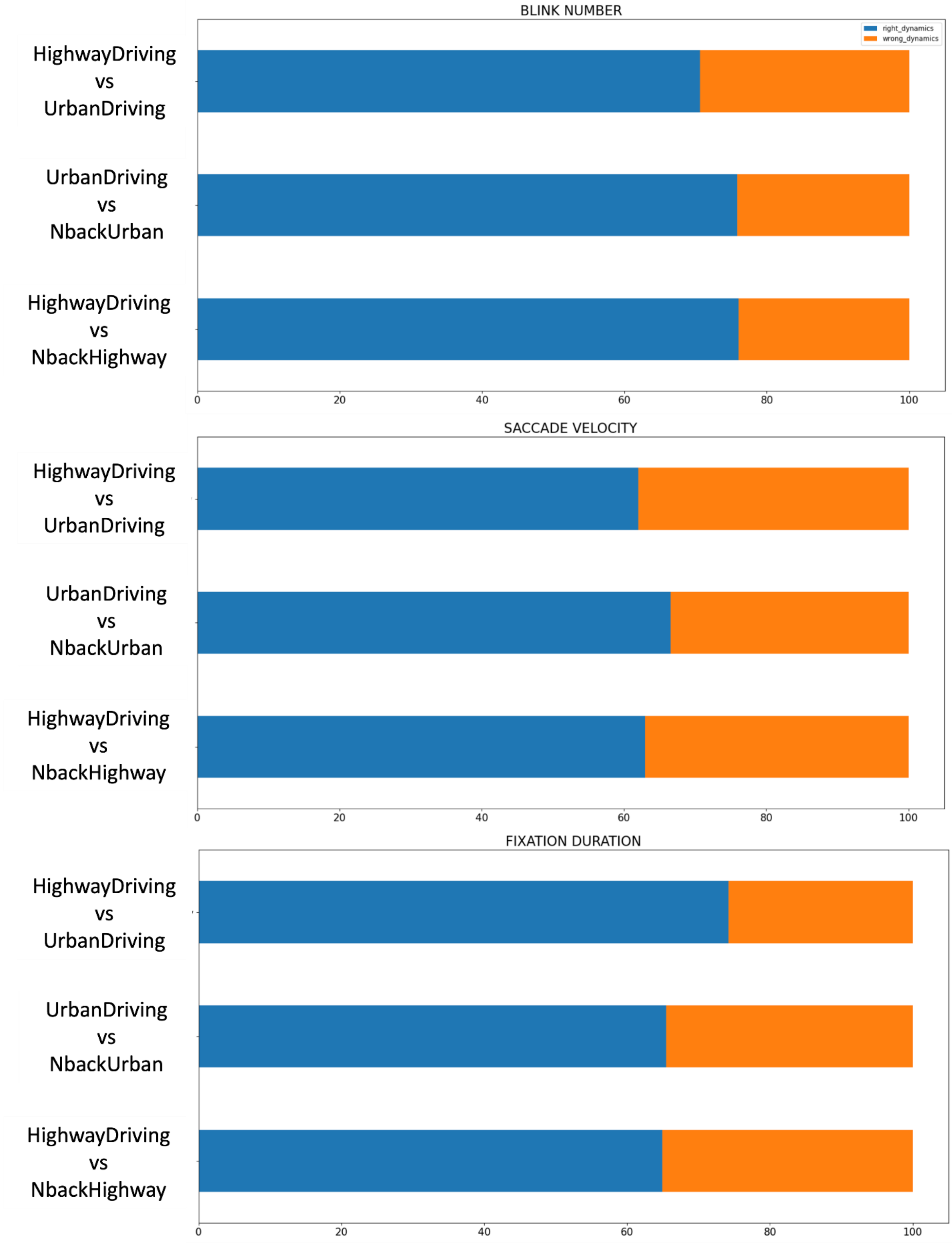

BLINK NUMBER
right_dynamics
wrong_dynamics
HighwayDriving vs UrbanDriving
UrbanDriving vs NbackUrban
HighwayDriving vs NbackHighway
0
20
40
60
80
100
SACCADE VELOCITY
HighwayDriving vs UrbanDriving
UrbanDriving vs NbackUrban
HighwayDriving vs NbackHighway
0
20
40
60
80
100
FIXATION DURATION
HighwayDriving vs UrbanDriving
UrbanDriving vs NbackUrban
HighwayDriving vs NbackHighway
0
20
40
60
80
100

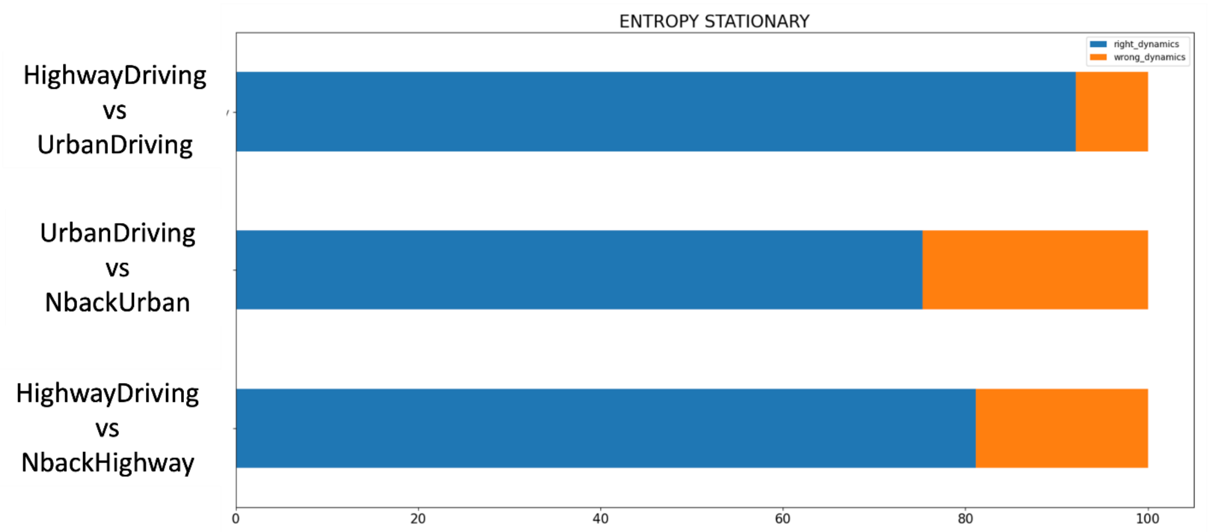


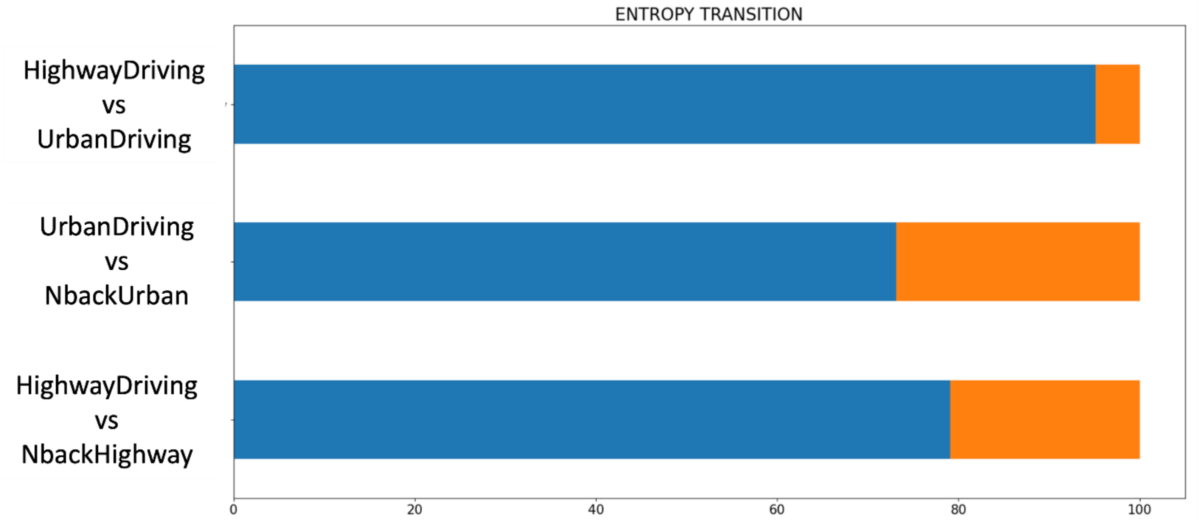


**Table S7. Self-reported cognitive load levels in NASA TLX scales.** Median and quartiles values of self-estimated values of cognitive load in experimental stages are shown along with Wilcoxon test results: * - p<0.01 in comparison of HighwayDriving and HighwayNback stages. & - p<0.01 in comparison of UrbanDriving and UrbanNback stages. # - p<0.01 in comparison of UrbanDriving and UrbanNback stages.

| NASA TLX scale | UrbanDriving | | | HighwayDriving | | | UrbanNback | | | HighwayNback | | | Wilcoxon test |
|---|---|---|---|---|---|---|---|---|---|---|---|---|---|
| | 25% | 50% | 75% | 25% | 50% | 75% | 25% | 50% | 75% | 25% | 50% | 75% | |
| **Effort** | -3 | 0 | 1 | -4 | -4 | -2 | 0 | 2 | 3 | -1 | 1 | 2 | * & # |
| **Temporal** | -3 | 0 | 1 | -4 | -4 | -3 | 0 | 2 | 3 | -1 | 1 | 2 | * & # |
| **Mental** | -4 | -2 | 1 | -4 | -4 | -3 | 2 | 3 | 4 | 0 | 2 | 3 | * & # |
| **Physical** | -4 | -2 | 1 | -4 | -4 | -3 | -2 | 0 | 2 | -4 | -2 | 0 | * & # |
| **Frustration** | -4 | -2 | 0 | -4 | -4 | -4 | -1 | 1 | 3 | -4 | -1.5 | 1 | * & # |
| **Performance** | 1 | 2 | 3 | 3 | 4 | 4 | -3 | -1 | 1 | -2 | 0 | 2 | * & # |
| **Averaged answer** | - 2.83 | - 1.08 | 0.29 | -4 | - 3.66 | - 2.66 | 0.33 | 1.33 | 2.16 | - 1.16 | 0 | 1 | * & # |

**Fig. S4. Percentages of subjects with expected difference in NASA TLX scale values between each pair of stages with higher and lower CL.**

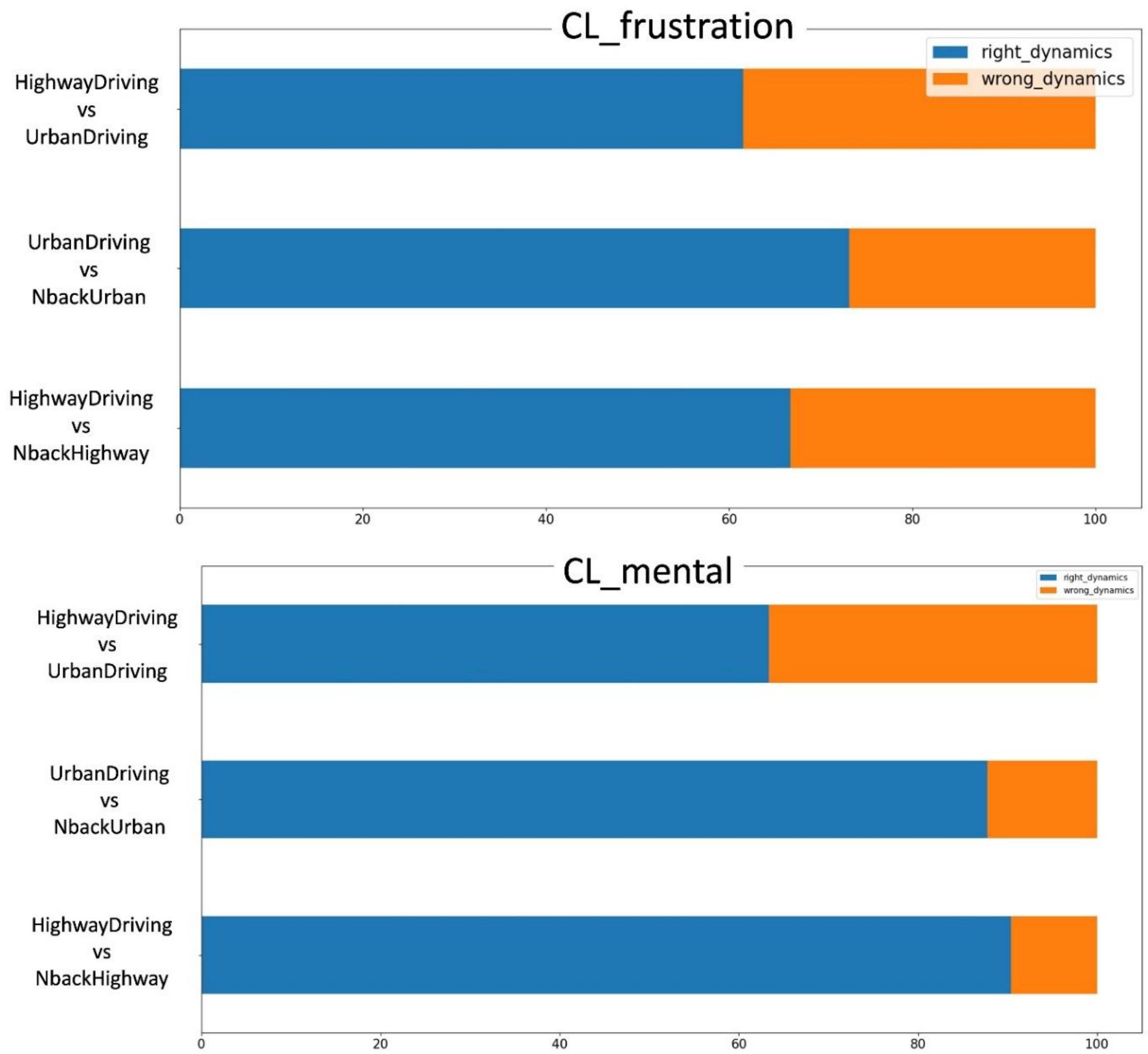

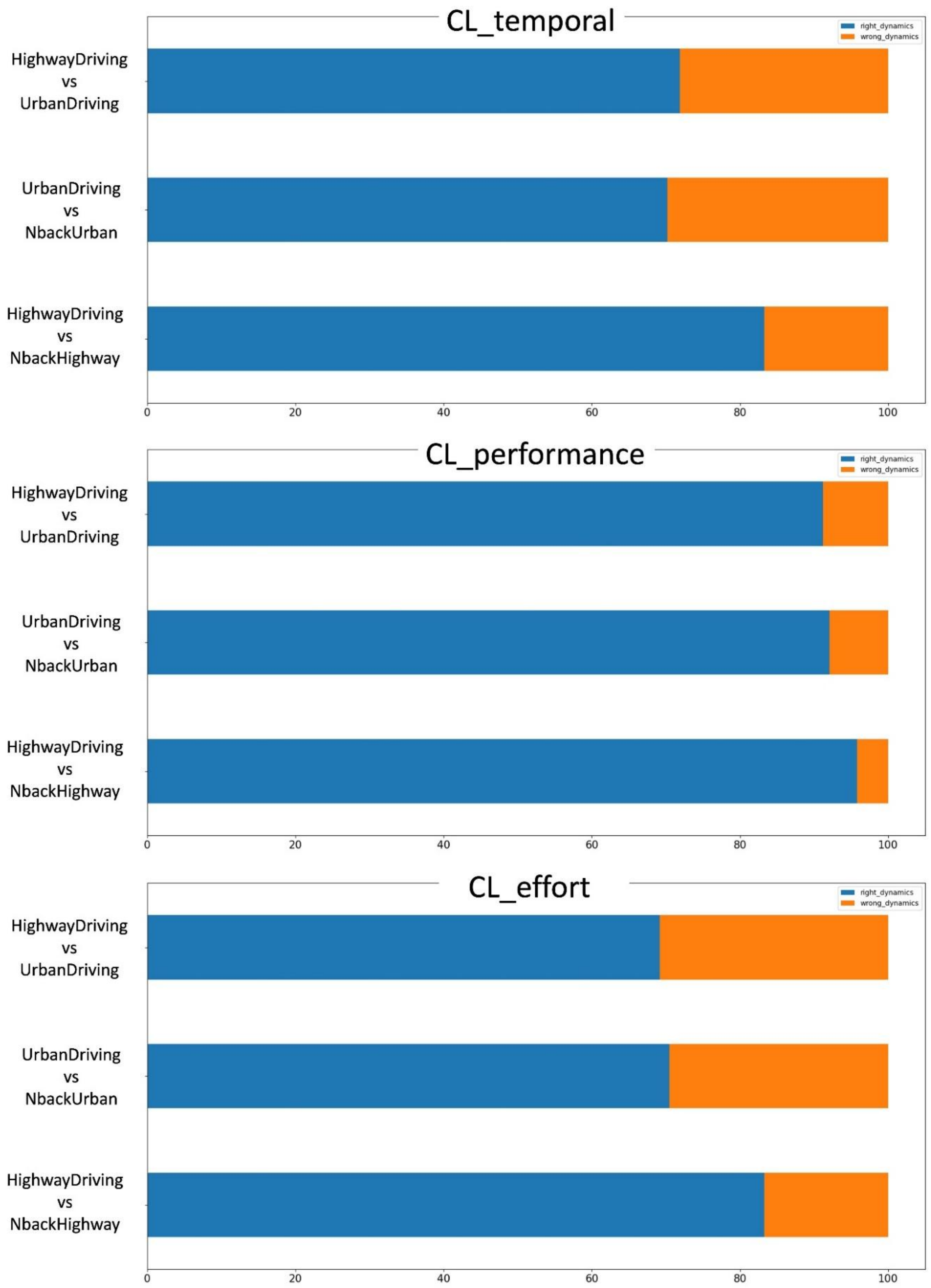
CL_temporal
right_dynamics
wrong_dynamics
HighwayDriving vs UrbanDriving
UrbanDriving vs NbackUrban
HighwayDriving vs NbackHighway
0
20
40
60
80
100
CL_performance
right_dynamics
wrong_dynamics
HighwayDriving vs UrbanDriving
UrbanDriving vs NbackUrban
HighwayDriving vs NbackHighway
0
20
40
60
80
100
CL_effort
right_dynamics
wrong_dynamics
HighwayDriving vs UrbanDriving
UrbanDriving vs NbackUrban
HighwayDriving vs NbackHighway
0
20
40
60
80
100

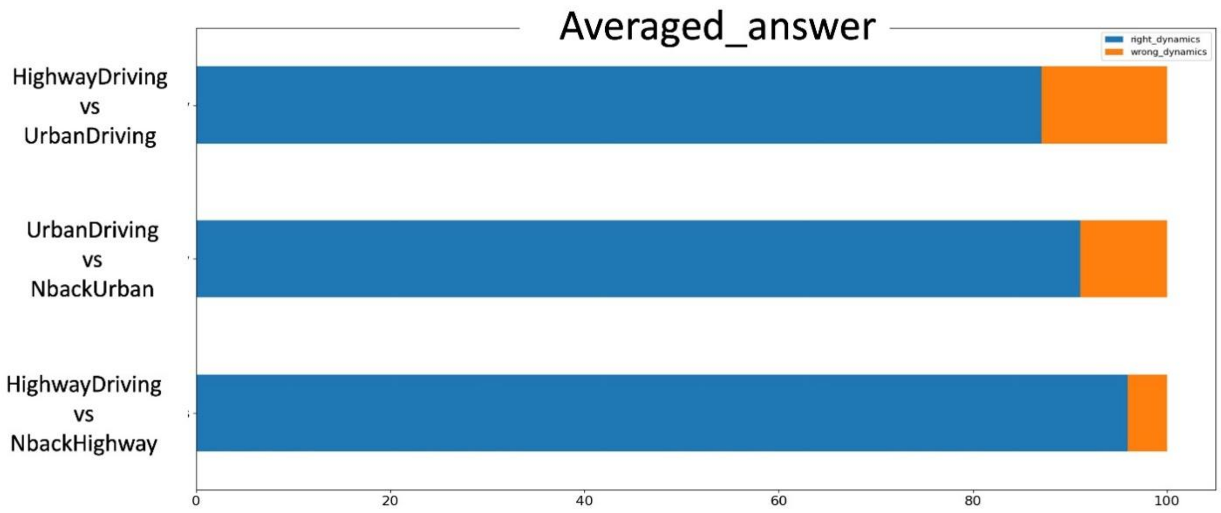


**Table S8. Correlation between eye gaze and self-report measures across all experimental stages.** Spearman rank correlation coefficients (r) and significance level (p) are shown for correlations between NASA TLX scales answers and eye gaze metrics, * p<0.01.

| NASA TLX scale | Eye gaze metric | r | p |
|---|---|---|---|
| CL_effort | Fixation_Duration | 0.014 | 0.45 |
| CL_effort | Saccade_velocity | -0.131 | 0.000* |
| CL_effort | Blink_number | 0.06 | 0.002* |
| CL_effort | SGE | 0.044 | 0.021 |
| CL_effort | GTE | -0.083 | 0.000* |
| CL_temporal | Fixation_Duration | -0.014 | 0.47 |
| CL_temporal | Saccade_velocity | -0.174 | 0.000* |
| CL_temporal | Blink_number | 0.038 | 0.04 |
| CL_temporal | SGE | 0.063 | 0.000* |
| CL_temporal | GTE | -0.108 | 0.000* |
| CL_mental | Fixation_Duration | 0.005 | 0.79 |
| CL_mental | Saccade_velocity | -0.183 | 0.000* |
| CL_mental | Blink_number | 0.095 | 0.000* |
| CL_mental | SGE | 0.013 | 0.48 |
| CL_mental | GTE | -0.068 | 0.000* |
| CL_physical | Fixation_Duration | -0.088 | 0.000* |
| CL_physical | Saccade_velocity | -0.174 | 0.000* |
| CL_physical | Blink_number | 0.032 | 0.09 |
| CL_physical | SGE | 0.132 | 0.000* |
| CL_physical | GTE | -0.183 | 0.000* |
| CL_frustration | Fixation_Duration | -0.072 | 0.000* |
| CL_frustration | Saccade_velocity | -0.155 | 0.000* |
| CL_frustration | Blink_number | 0.043 | 0.02 |

| | | | |
|---|---|---|---|
| CL_frustration | SGE | 0.125 | 0.000* |
| CL_frustration | GTE | -0.186 | 0.000* |
| CL_performance | Fixation_Duration | -0.065 | 0.000* |
| CL_performance | Saccade_velocity | -0.166 | 0.000* |
| CL_performance | Blink_number | 0.102 | 0.000* |
| CL_performance | SGE | 0.041 | 0.03 |
| CL_performance | GTE | -0.131 | 0.000* |
| Averaged answer | Fixation_Duration | -0.022 | 0.25 |
| Averaged answer | Saccade_velocity | -0.179 | 0.000* |
| Averaged answer | Blink_number | 0.046 | 0.015 |
| Averaged answer | SGE | 0.09 | 0.000* |
| Averaged answer | GTE | -0.131 | 0.000* |

**Table S9. Correlation between eye gaze and self-report measures for each experimental stage separately.** Spearman coefficients (r) and significance level (p) are shown for correlations between NASA TLX responses and eye gaze metrics, * p<0.01.

| Experimental stage | NASA TLX scale | Eye-gaze metric | r | p |
|---|---|---|---|---|
| HighwayDriving | CL_effort | Fixation_Duration | 0.003 | 0.93 |
| HighwayDriving | CL_effort | Saccade_velocity | -0.02 | 0.48 |
| HighwayDriving | CL_effort | Blink_number | -0.03 | 0.39 |
| HighwayDriving | CL_effort | SGE | -0.01 | 0.87 |
| HighwayDriving | CL_effort | GTE | -0.01 | 0.71 |
| HighwayDriving | CL_temporal | Fixation_Duration | -0.06 | 0.10 |
| HighwayDriving | CL_temporal | Saccade_velocity | -0.05 | 0.18 |
| HighwayDriving | CL_temporal | Blink_number | -0.06 | 0.11 |
| HighwayDriving | CL_temporal | SGE | 0.03 | 0.35 |
| HighwayDriving | CL_temporal | GTE | -0.04 | 0.31 |
| HighwayDriving | CL_mental | Fixation_Duration | -0.03 | 0.45 |
| HighwayDriving | CL_mental | Saccade_velocity | -0.09 | 0.02 |
| HighwayDriving | CL_mental | Blink_number | -0.06 | 0.14 |
| HighwayDriving | CL_mental | SGE | 0.001 | 0.97 |
| HighwayDriving | CL_mental | GTE | -0.02 | 0.48 |
| HighwayDriving | CL_physical | Fixation_Duration | -0.02 | 0.56 |
| HighwayDriving | CL_physical | Saccade_velocity | -0.09 | 0.01 |
| HighwayDriving | CL_physical | Blink_number | -0.04 | 0.31 |
| HighwayDriving | CL_physical | SGE | 0.01 | 0.87 |
| HighwayDriving | CL_physical | GTE | -0.04 | 0.28 |
| HighwayDriving | CL_frustration | Fixation_Duration | 0.02 | 0.61 |
| HighwayDriving | CL_frustration | Saccade_velocity | 0.03 | 0.43 |

| | | | | |
|---|---|---|---|---|
| HighwayDriving | CL_frustration | Blink_number | -0.03 | 0.50 |
| HighwayDriving | CL_frustration | SGE | 0.03 | 0.40 |
| HighwayDriving | CL_frustration | GTE | 0.02 | 0.59 |
| HighwayDriving | CL_performance | Fixation_Duration | -0.09 | 0.01 |
| HighwayDriving | CL_performance | Saccade_velocity | -0.02 | 0.53 |
| HighwayDriving | CL_performance | Blink_number | 0.04 | 0.31 |
| HighwayDriving | CL_performance | SGE | 0.01 | 0.76 |
| HighwayDriving | CL_performance | GTE | -0.06 | 0.10 |
| HighwayDriving | Averaged answer | Fixation_Duration | 0.01 | 0.75 |
| HighwayDriving | Averaged answer | Saccade_velocity | -0.06 | 0.11 |
| HighwayDriving | Averaged answer | Blink_number | -0.06 | 0.12 |
| HighwayDriving | Averaged answer | SGE | -0.01 | 0.86 |
| HighwayDriving | Averaged answer | GTE | 0.01 | 0.87 |
| HighwayNback | CL_effort | Fixation_Duration | 0.03 | 0.42 |
| HighwayNback | CL_effort | Saccade_velocity | 0.05 | 0.23 |
| HighwayNback | CL_effort | Blink_number | -0.02 | 0.55 |
| HighwayNback | CL_effort | SGE | -0.04 | 0.31 |
| HighwayNback | CL_effort | GTE | -0.02 | 0.68 |
| HighwayNback | CL_temporal | Fixation_Duration | 0.03 | 0.44 |
| HighwayNback | CL_temporal | Saccade_velocity | 0.05 | 0.23 |
| HighwayNback | CL_temporal | Blink_number | -0.04 | 0.32 |
| HighwayNback | CL_temporal | SGE | -0.04 | 0.28 |
| HighwayNback | CL_temporal | GTE | -0.01 | 0.74 |
| HighwayNback | CL_mental | Fixation_Duration | 0.02 | 0.55 |
| HighwayNback | CL_mental | Saccade_velocity | 0.004 | 0.91 |
| HighwayNback | CL_mental | Blink_number | -0.04 | 0.35 |
| HighwayNback | CL_mental | SGE | -0.03 | 0.44 |
| HighwayNback | CL_mental | GTE | -0.03 | 0.41 |
| HighwayNback | CL_physical | Fixation_Duration | -0.04 | 0.34 |
| HighwayNback | CL_physical | Saccade_velocity | -0.03 | 0.41 |
| HighwayNback | CL_physical | Blink_number | 0.02 | 0.66 |
| HighwayNback | CL_physical | SGE | -0.01 | 0.77 |
| HighwayNback | CL_physical | GTE | -0.05 | 0.21 |
| HighwayNback | CL_frustration | Fixation_Duration | -0.03 | 0.46 |
| HighwayNback | CL_frustration | Saccade_velocity | 0.003 | 0.93 |
| HighwayNback | CL_frustration | Blink_number | 0.004 | 0.92 |
| HighwayNback | CL_frustration | SGE | -0.05 | 0.16 |
| HighwayNback | CL_frustration | GTE | -0.08 | 0.03 |
| HighwayNback | CL_performance | Fixation_Duration | -0.06 | 0.13 |

| | | | | |
|---|---|---|---|---|
| HighwayNback | CL_performance | Saccade_velocity | 0.01 | 0.86 |
| HighwayNback | CL_performance | Blink_number | 0.01 | 0.87 |
| HighwayNback | CL_performance | SGE | -0.06 | 0.12 |
| HighwayNback | CL_performance | GTE | -0.07 | 0.08 |
| HighwayNback | Averaged answer | Fixation_Duration | 0.02 | 0.58 |
| HighwayNback | Averaged answer | Saccade_velocity | 0.01 | 0.75 |
| HighwayNback | Averaged answer | Blink_number | -0.02 | 0.61 |
| HighwayNback | Averaged answer | SGE | -0.03 | 0.41 |
| HighwayNback | Averaged answer | GTE | -0.03 | 0.37 |
| UrbanDriving | CL_effort | Fixation_Duration | 0.04 | 0.29 |
| UrbanDriving | CL_effort | Saccade_velocity | -0.01 | 0.83 |
| UrbanDriving | CL_effort | Blink_number | 0.13 | 0.000* |
| UrbanDriving | CL_effort | SGE | -0.09 | 0.02 |
| UrbanDriving | CL_effort | GTE | 0.03 | 0.42 |
| UrbanDriving | CL_temporal | Fixation_Duration | 0.02 | 0.56 |
| UrbanDriving | CL_temporal | Saccade_velocity | -0.06 | 0.11 |
| UrbanDriving | CL_temporal | Blink_number | 0.09 | 0.01 |
| UrbanDriving | CL_temporal | SGE | -0.11 | 0.005 |
| UrbanDriving | CL_temporal | GTE | 0.04 | 0.27 |
| UrbanDriving | CL_mental | Fixation_Duration | -0.02 | 0.61 |
| UrbanDriving | CL_mental | Saccade_velocity | -0.07 | 0.07 |
| UrbanDriving | CL_mental | Blink_number | 0.11 | 0.007 |
| UrbanDriving | CL_mental | SGE | -0.16 | 0.000* |
| UrbanDriving | CL_mental | GTE | 0.03 | 0.43 |
| UrbanDriving | CL_physical | Fixation_Duration | -0.04 | 0.31 |
| UrbanDriving | CL_physical | Saccade_velocity | -0.08 | 0.04 |
| UrbanDriving | CL_physical | Blink_number | 0.17 | 0.000* |
| UrbanDriving | CL_physical | SGE | -0.14 | 0.000* |
| UrbanDriving | CL_physical | GTE | 0.001 | 0.98 |
| UrbanDriving | CL_frustration | Fixation_Duration | -0.05 | 0.22 |
| UrbanDriving | CL_frustration | Saccade_velocity | -0.04 | 0.29 |
| UrbanDriving | CL_frustration | Blink_number | 0.13 | 0.000* |
| UrbanDriving | CL_frustration | SGE | -0.13 | 0.000* |
| UrbanDriving | CL_frustration | GTE | 0.001 | 0.97 |
| UrbanDriving | CL_performance | Fixation_Duration | -0.05 | 0.19 |
| UrbanDriving | CL_performance | Saccade_velocity | -0.04 | 0.32 |
| UrbanDriving | CL_performance | Blink_number | 0.09 | 0.01 |
| UrbanDriving | CL_performance | SGE | -0.11 | 0.005 |
| UrbanDriving | CL_performance | GTE | -0.04 | 0.31 |

| | | | | |
|---|---|---|---|---|
| UrbanDriving | Averaged answer | Fixation_Duration | 0.01 | 0.88 |
| UrbanDriving | Averaged answer | Saccade_velocity | -0.06 | 0.11 |
| UrbanDriving | Averaged answer | Blink_number | 0.14 | 0.000* |
| UrbanDriving | Averaged answer | SGE | -0.14 | 0.000* |
| UrbanDriving | Averaged answer | GTE | 0.05 | 0.24 |
| UrbanNback | CL_effort | Fixation_Duration | 0.05 | 0.17 |
| UrbanNback | CL_effort | Saccade_velocity | 0.05 | 0.24 |
| UrbanNback | CL_effort | Blink_number | 0.01 | 0.83 |
| UrbanNback | CL_effort | SGE | -0.12 | 0.001* |
| UrbanNback | CL_effort | GTE | 0.02 | 0.56 |
| UrbanNback | CL_temporal | Fixation_Duration | 0.05 | 0.19 |
| UrbanNback | CL_temporal | Saccade_velocity | -0.02 | 0.56 |
| UrbanNback | CL_temporal | Blink_number | 0.06 | 0.14 |
| UrbanNback | CL_temporal | SGE | -0.15 | 0.000* |
| UrbanNback | CL_temporal | GTE | 0.05 | 0.21 |
| UrbanNback | CL_mental | Fixation_Duration | -0.02 | 0.71 |
| UrbanNback | CL_mental | Saccade_velocity | 0.07 | 0.05 |
| UrbanNback | CL_mental | Blink_number | 0.06 | 0.09 |
| UrbanNback | CL_mental | SGE | -0.05 | 0.16 |
| UrbanNback | CL_mental | GTE | -0.06 | 0.11 |
| UrbanNback | CL_physical | Fixation_Duration | -0.05 | 0.21 |
| UrbanNback | CL_physical | Saccade_velocity | -0.03 | 0.37 |
| UrbanNback | CL_physical | Blink_number | 0.07 | 0.05 |
| UrbanNback | CL_physical | SGE | -0.01 | 0.72 |
| UrbanNback | CL_physical | GTE | -0.03 | 0.38 |
| UrbanNback | CL_frustration | Fixation_Duration | -0.06 | 0.11 |
| UrbanNback | CL_frustration | Saccade_velocity | 0.04 | 0.32 |
| UrbanNback | CL_frustration | Blink_number | 0.09 | 0.02 |
| UrbanNback | CL_frustration | SGE | -0.08 | 0.04 |
| UrbanNback | CL_frustration | GTE | -0.07 | 0.08 |
| UrbanNback | CL_performance | Fixation_Duration | -0.14 | 0.000* |
| UrbanNback | CL_performance | Saccade_velocity | 0.05 | 0.24 |
| UrbanNback | CL_performance | Blink_number | 0.12 | 0.002* |
| UrbanNback | CL_performance | SGE | -0.02 | 0.61 |
| UrbanNback | CL_performance | GTE | -0.18 | 0.000* |
| UrbanNback | Averaged answer | Fixation_Duration | 0.03 | 0.46 |
| UrbanNback | Averaged answer | Saccade_velocity | -0.01 | 0.87 |
| UrbanNback | Averaged answer | Blink_number | 0.04 | 0.24 |
| UrbanNback | Averaged answer | SGE | -0.12 | 0.001* |

| | | | | |
|---|---|---|---|---|
| UrbanNback | Averaged answer | GTE | 0.04 | 0.33 |

**Table S10. Absolute coherence values between stages, eye gaze metrics and NASA TLX responses in pairs of stages with different levels of CL.** Chi-square test, * - p< 0.01.

| Experiment stages | NBACK TLX scales | Blink number | SGE | GTE | Fixation duration | Saccade velocity | Saccade velocity |
|---|---|---|---|---|---|---|---|
| **UrbanDriving vs UrbanNback** | CL_effort | 0.53* | 0.64* | 0.51 | 0.51 | 0.66* | 0.58* |
| | CL_temporal | 0.52 | 0.64* | 0.51 | 0.51 | 0.66* | 0.60* |
| | CL_mental | 0.58* | 0.65* | 0.55* | 0.51 | 0.67* | 0.59* |
| | CL_physical | 0.51 | 0.62* | 0.53* | 0.56* | 0.67* | 0.60* |
| | CL_frustration | 0.54* | 0.64* | 0.50 | 0.53* | 0.67* | 0.59* |
| | CL_performance | 0.56* | 0.64* | 0.50 | 0.53* | 0.57* | 0.58* |
| | Averaged answer | 0.58* | 0.61* | 0.55* | 0.52 | 0.65* | 0.56* |
| | **average** | **0.55*** | **0.63*** | **0.52** | **0.52** | **0.65*** | **0.58*** |
| **HighwayDriving vs HighwayNback** | CL_effort | 0.54* | 0.68* | 0.59* | 0.53* | 0.62* | 0.63* |
| | CL_temporal | 0.53* | 0.70* | 0.58* | 0.52 | 0.63* | 0.64* |
| | CL_mental | 0.56* | 0.70* | 0.62* | 0.55* | 0.63* | 0.64* |
| | CL_physical | 0.58* | 0.71* | 0.57* | 0.60* | 0.68* | 0.70* |
| | CL_frustration | 0.55* | 0.74* | 0.52 | 0.56* | 0.68* | 0.69* |
| | CL_performance | 0.56* | 0.70* | 0.60* | 0.52 | 0.55* | 0.64* |
| | Averaged answer | 0.57* | 0.65* | 0.62* | 0.56* | 0.63* | 0.60* |
| | **average** | **0.56*** | **0.70*** | **0.58*** | **0.55*** | **0.63*** | **0.65*** |
| **HighwayDriving vs UrbanDriving** | CL_effort | 0.63* | 0.61* | 0.76* | 0.65* | 0.75* | 0.67* |
| | CL_temporal | 0.66* | 0.62* | 0.80* | 0.68* | 0.78* | 0.69* |
| | CL_mental | 0.67* | 0.56* | 0.80* | 0.70* | 0.79* | 0.71* |
| | CL_physical | 0.66* | 0.52 | 0.78* | 0.69* | 0.78* | 0.71* |
| | CL_frustration | 0.70* | 0.54* | 0.84* | 0.73* | 0.82* | 0.73* |
| | CL_performance | 0.65* | 0.59* | 0.80* | 0.69* | 0.59* | 0.70* |
| | Averaged answer | 0.59* | 0.68* | 0.73* | 0.61* | 0.63* | 0.63* |
| | **average** | **0.65*** | **0.59*** | **0.79*** | **0.68*** | **0.73*** | **0.69*** |
